# Comparative Genomics of *Cryptosporidium*

**DOI:** 10.1155/2013/832756

**Published:** 2013-05-02

**Authors:** Aurélien J. Mazurie, João M. Alves, Luiz S. Ozaki, Shiguo Zhou, David C. Schwartz, Gregory A. Buck

**Affiliations:** ^1^Department of Microbiology, Montana State University, Bozeman, MT 59717, USA; ^2^Department of Microbiology and Immunology, Virginia Commonwealth University, Richmond, VA 23284-2030, USA; ^3^Laboratory for Molecular and Computational Genomics, Department of Chemistry, Laboratory of Genetics, University of Wisconsin-Madison, Madison, WI 53706, USA

## Abstract

Until recently, the apicomplexan parasites, *Cryptosporidium hominis* and *C. parvum*, were considered the same species. However, the two parasites, now considered distinct species, exhibit significant differences in host range, infectivity, and pathogenicity, and their sequenced genomes exhibit only 95–97% identity. The availability of the complete genome sequences of these organisms provides the potential to identify the genetic variations that are responsible for the phenotypic differences between the two parasites. We compared the genome organization and structure, gene composition, the metabolic and other pathways, and the local sequence identity between the genes of these two *Cryptosporidium* species. Our observations show that the phenotypic differences between *C. hominis* and *C. parvum* are not due to gross genome rearrangements, structural alterations, gene deletions or insertions, metabolic capabilities, or other obvious genomic alterations. Rather, the results indicate that these genomes exhibit a remarkable structural and compositional conservation and suggest that the phenotypic differences observed are due to subtle variations in the sequences of proteins that act at the interface between the parasite and its host.

## 1. Introduction

Organisms of the genus *Cryptosporidium* are protozoa of the phylum Apicomplexa. These obligatory intracellular organisms parasitize animals of all vertebrate classes [1]. Although mostly ignored as a pathogen until relatively late in the 20th century, diarrhea caused by *Cryptosporidium* species is debilitating for adults and children and can be life threatening for immunocompromised individuals such as those with AIDS. Cryptosporidiosis is also a significant factor in animal husbandry practices and represents a significant challenge to agriculture, for example, the beef industry [2]. Development of molecular tools now permits efficient differentiation of morphologically indistinguishable isolates of these parasites, and this new capability has led to important new insights into their epidemiology and pathogenicity. Although several *Cryptosporidium* species can cause human disease, two species, *C. hominis* and *C. parvum*, are responsible for the majority of the human impact. *C. parvum* infects ruminants as primary hosts and humans as incidental hosts. *C. hominis*, in contrast, is highly infectious to humans but generally does not infect other species [3]. Until very recently, these two species were considered genotypes of *C. parvum* [4]: genotype 1 (or type H) found nearly exclusively in humans; and genotype 2 (or type C) found naturally infecting cattle and other animals as well as humans [5]. Later investigations established that these genotypes are sufficiently distinct in host range, genetics, pathogenicity, intensity of infection, and other growth characteristics to be considered separate species [6]. Recently, it has been shown that these two parasites use different mechanisms for host cell invasion, a significant finding considering their differential host preferences [7].

Cryptosporidiosis is a zoonotic, primarily water-borne disease that is transmitted by the oral-fecal route. *Cryptosporidium* has a simpler life cycle than other apicomplexans. The only life stage found outside the host is the oocyst, a resistant spore-like form that is largely quiescent until ingested by a new host. Although the disease is usually self-limiting, it has been suggested that cryptosporidiosis can be a significant component in malnutrition, impaired growth, and intellectual acuity in developing countries, where children are exposed to repeated infections during a critical time of their development [8]. In developed countries, drinking and recreational water-borne outbreaks and their economical consequences, as well as agricultural and veterinary impacts are the major concerns. Immunocompromised individuals and the elderly are also at risk of complications of cryptosporidiosis, since immunoprophylactics for the disease are unavailable, and treatment is often late and targeted at symptoms.

With the completion of the genome sequences of *C. hominis* and *C. parvum* [9, 10], it is now possible to carefully and accurately compare their genetic architectures and compositions with the goal of identifying the root causes of their phenotypic differences. Herein, comparisons were performed at three levels. First, the genome sequences were compared, focusing on general features of genome organization, for example, rearrangements and insertions or deletions. Second, gene level comparisons were performed with two goals: to evaluate the gene complements and compositions of these organisms and to search for specific genes undergoing noticeable selective pressure, as determined by nonsynonymous to synonymous substitution ratios in protein evolution. Finally, comparisons at the level of inferred pathways were performed to investigate how eventual differences in gene composition could impact the organization of metabolic and other networks in these organisms. Thus, genomes of the two species of *Cryptosporidium* were carefully compared to each other and simultaneously to the genomes of other apicomplexans for which the genomes are available.

## 2. Results and Discussion

### 2.1. Genome Synteny and Collinearity

To analyze the genomic organization in these two organisms, we used the published *C. parvum* sequence [10], updated with additional data, and an updated assembly of the published *C. hominis* genome [9]. In the new *C. hominis* assembly (deposited at DDBJ/EMBL/GenBank under the whole genome shotgun project accession number AAEL00000000, the version used in this paper being AAEL02000000), additional directed sequencing was used to close most of the sequence gaps and reduce the number of contigs to ~330 with an N_50_ of 49.2 kb. Alignment of the *C. hominis* and *C. parvum* contigs revealed no significant differences; that is, despite the 3–5% sequence variation observed between the two genomes, we detected no significant insertions, deletions, or rearrangements.

This observation was confirmed by generation of genome wide optical maps of both species. Thus, optical maps of the *C. hominis* genome generated using the SacI restriction enzyme were compared to the virtual SacI restriction maps of the genome sequences of *C. parvum* (see Section 4). Discrepancies were found in the ordering and orientation of contigs of the two species. Each of these apparent discrepancies was examined by attempting to amplify the DNA from both *C. hominis* and *C. parvum* in the apparent gaps using primers targeting the ends of the contigs in question in polymerase chain reaction (PCR) experiments (see Section 4). These experiments confirmed that apparent organizational discrepancies, for example, rearrangements, between the two genomes were due to incorrect assemblies (data not shown). For example, the orientations of fragment no. 1 of chromosome 2 and fragment no. 2 of chromosome 4 in the published *C. parvum* sequence [10] are reversed. No insertions or deletions of significant size were detected, and most differences in chromosome length were due to remaining gaps in the *C. hominis* sequence. In summary, despite the fairly high level of sequence divergence observed between *C. hominis* and *C. parvum*, the genomes of these two parasites are essentially collinear. Since both parasites exhibit the same gene composition (see below), we conclude they are syntenic at all sites in all eight of their chromosomes.

The synteny between the *C. parvum* genome and those of three other apicomplexans, selected for the quality of their genome annotation (*Plasmodium falciparum*, *P. knowlesi*, and *P. vivax*), was evaluated by comparing both chromosome composition and gene order. Thus, orthologs were identified and each chromosome of the three plasmodia species was evaluated for its enrichment in orthologs from given chromosomes of *C. parvum* (see Section 4). As shown in Table S1 in Supplementary Material available online at http://dx.doi.org/10.1155/2013/832756, orthologs are not uniformly spread into the plasmodia chromosomes. Thus, although the genes from each *Cryptosporidium* chromosome are dispersed among the *Plasmodium* chromosomes, a significant fraction of them are colocalized on a given *Plasmodium* chromosome. This observation suggests that chromosome composition is significantly conserved across the apicomplexans. However, despite this conservation of gene composition, the order of the genes on the chromosomes (i.e., the synteny) shows little evidence of conservation between the *Cryptosporidium* and plasmodia (see Supplementary Figure 1). These results strongly suggest that genomes of these parasites evolved from a common ancestor through extensive *cis*-rearrangements (insertion, deletion, and reordering of genome fragments or genes within chromosomes) rather than through *trans*-rearrangements (exchange of genome fragments or genes among chromosomes).

We have previously reported that the genome of *Cryptosporidium* is heavily tailored and apparently has maintained only the fraction of genes required for its survival in the specific environments it inhabits [9, 10]. In contrast, the genomes of other apicomplexans, including the *Plasmodium* species, are quite robust. Thus, we assume that the genome of the apicomplexan progenitor was more robust and that the *Cryptosporidium* genome is the product of broad-scale gene deletion. Although the selective pressures for this phenomena are apparent, the genetic mechanisms remain obscure. It is interesting therefore to note that the mechanism that led to broad scale deletions in *Cryptosporidium* largely preserved the gene compositions of specific chromosome fragments while simultaneously not conserving the gene order.

### 2.2. Gene Complement and Composition

We compared the gene complements of *C. hominis* and *C. parvum* in order to investigate potential differences in the repertoires of proteins encoded. We found that effectively all genes from one organism have a corresponding putative ortholog in the other. The only apparent exceptions were 67 genes initially found only in *C. parvum* and 246 genes initially present only in *C. hominis*. However, more careful alignment of these genes to the corresponding genome [11] showed that all putative *C. parvum*-specific genes are also present in *C. hominis* but were not predicted due to very small gaps remaining in the sequence. Conversely, all but one of the genes apparently specific to *C. hominis* were similarly found in the *C. parvum* genome. The remaining apparently *C. hominis*-specific gene (Chro.00003) is truly absent in *C. parvum*. This gene is in a contig that is unlinked to the remainder of the *C. hominis* genome, and its sequence most closely resembles related genes of alphaproteobacterial origin (data not shown). Since *C. hominis* is purified from the feces of an infected mammal, this single remaining gene is most likely derived from an alphaproteobacterial contaminant of the initial *C. hominis* stock used to generate shotgun sequence for the *C. hominis* genome. Therefore, we conclude that *C. hominis* and *C. parvum* have identical gene complements.

We also examined the frequency of putative paralogs per gene across all Apicomplexa for which the genomes are available (Supplementary Figure 2). This analysis showed that *Cryptosporidium* has less than 0.05 paralogs per gene, while the next most compact genomes (*Plasmodium berghei* and *Toxoplasma gondii*) have levels of gene duplication about one order of magnitude higher (0.43 and 0.53, resp.). Species of *Theileria* exhibit 0.8–1.4 paralogs per gene, despite having genomes about 1 Mb smaller than *Cryptosporidium* and a similar number of genes (~3,800 to ~4,000) [12]. In short, our analysis suggested that *Cryptosporidium* species, in spite of having neither the smallest genomes nor the lowest numbers of proteins, have the most compact proteomes of these apicomplexans, exhibiting the lowest level of redundancy. Clearly, such a low level of protein redundancy is convenient for the investigation of potential drug targets and vaccine candidates for treating and preventing cryptosporidiosis.

### 2.3. Metabolic and Signaling Capabilities

**Figure 1:**
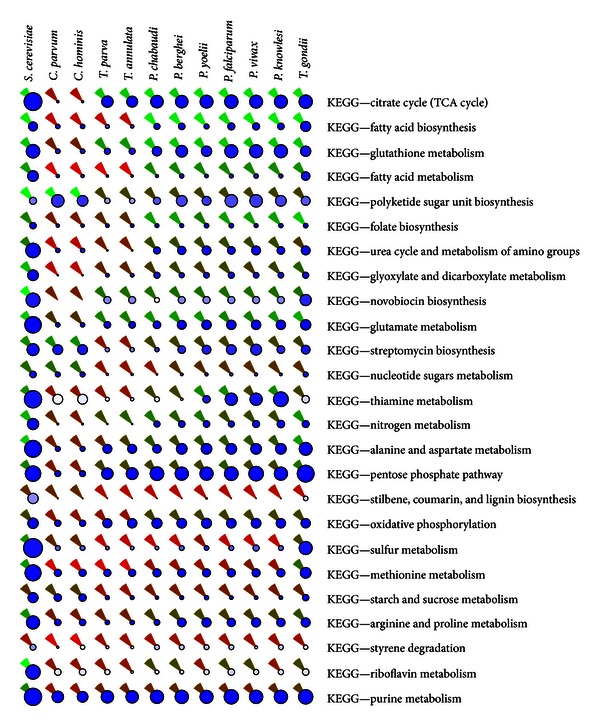
Pathway scores (excerpt). Completeness, connectedness, and support scores of the inferred metabolic pathways of *C. hominis* and *C. parvum*, along with nine other apicomplexans and an external reference (*Saccharomyces cerevisiae*). The three scores are represented as follows. The color of the circles reflects the support, from white (support of 0) to blue (maximal support). The size of each circle is proportional to the completeness; that is, the larger the circle, the more complete the pathway (see text). The color of the wedge—available for metabolic pathways only—reflects the connectedness, from red (connectedness of 0) to green (connectedness of 100%). Species are clustered according to their completeness score. Pathways are ranked by decreasing power to discriminate between the *Cryptosporidium* and the other apicomplexans. Only the 25 most discriminative pathways are shown; a complete figure is available as Supplementary Figure 3. All score values are available in Supplementary Table 2.

Metabolic and signaling pathways were identified in the two *Cryptosporidium* species and other apicomplexans for which sequences are available, using sequence similarity approaches (see Section 4). In order to evaluate any potential impairment of these pathways due to the minor gene content discrepancies identified above, we evaluated the pathways using three scores: completeness, connectedness, and support (Figure 1). These scores, described in Section 4, reflect how complete the enzymatic equipment of a given pathway is, as well as the ability of the pathway to process key metabolites shared with other pathways [13, 14]. A high completeness score for a pathway indicates that most of the genes that are traditionally associated with this pathway are present in the genome. A high connectedness score indicates that the actual set of enzymes present ensures the interconversion of the metabolites, both input and output, that the pathway exchanges with other pathways. Finally, the support score reflects the number of reference species from the pathway databases that have been used to assess the presence of this pathway in *Cryptosporidium*. Therefore, a high support score reflects a high level of confidence that the annotation of the pathway is correct.

Figure 1—an excerpt of Supplementary Figure 3—provides a comparative analysis of *Cryptosporidium* and nine other apicomplexans for which genomic data is available (*T. parva*, *T. annulata*, *Plasmodium chabaudi*, *P. berghei*, *P. yoelii*, *P. falciparum*, *P. vivax*, *P. knowlesi*, and *Toxoplasma gondii*). The figure is a composite graphical representation of the three scores—completeness, connectedness, and support—for the two species of *Cryptosporidium*, the nine other apicomplexans, and an external reference (*S. cerevisiae*). The 12 species were clustered according to the completeness of their pathways. All scores are available in Supplementary Table 2.

As expected from the comparative analyses outlined above, these results confirmed that the two *Cryptosporidium* species have highly similar pathways and other functions. In contrast, their metabolic potential is markedly distinct from the other Apicomplexa. Despite the fact that some of the other species in the phylum have smaller genomes (e.g., *Theileria* spp. genomes are about 1 Mb smaller than the ~9.2 Mb genomes of the *Cryptosporidium* spp.), *Cryptosporidium* has the most highly reduced metabolic capabilities. As previously reported [9], and in contrast to most other apicomplexans, our observations further confirm that *Cryptosporidium* lacks most mitochondrial and apicoplast functions. Biosynthetic capabilities (amino acid, nucleic acid, carbohydrate, etc.) are limited, and energy generation machinery is largely dependent on glycolysis as the TCA cycle, oxidative phosphorylation, pentose phosphate pathway, and so forth are largely absent. This diminished metabolic capability is likely related to the relatively simple life cycle of the parasite; that is, in contrast to most other apicomplexan parasites, *Cryptosporidium* has only a single host and no vector. Clearly, the parasite is highly evolved to take advantage of the host functions and capabilities. For example, the *C. hominis* genome encodes more than 80 predicted transporters [9], while the similarly sized genome of *T. parva* seems to encode only about 60 transporters [15]. Another important difference is the giant enzyme, bacterial derived type I fatty acid biosynthesis mechanism present in *Cryptosporidium* [16], in contrast with the multienzyme type II mechanism functional in the apicoplast of all other Apicomplexa.

### 2.4. Gene Sequence Comparison

The high degree of identity of the gene compositions and pathways of *C. hominis* and *C. parvum* begs the question of the root of their clear phenotypic differences. Lack of differences in gene composition suggests a more subtle cause, for example, missense mutations in protein coding sequences, for the differences in their characteristics. The ratio of nonsynonymous missense and synonymous substitution rates (*dN*/*dS* ratio, see Section 4) is often used to identify genes subject to positive or negative selection within a given genome. Nonsynonymous changes in a DNA sequence, that is, mutations that change the amino acid sequence, are more likely to alter protein function and would therefore be expected to be rapidly eliminated from essential or important functional genes in microbial genomes. Genes with a *dN*/*dS* ratio greater than 1.0 are generally considered to be selected for sequence change (positive selection), while genes with a ratio below 1.0 are considered to be selected for sequence conservation (negative selection). We calculated *dN*/*dS* ratios for the pairs of orthologs in *C. hominis* and *C. parvum* as identified above to assess which genes were being selected for change or driven to evolve and those that are highly conserved in an attempt to begin identifying the source of the phenotypic divergence between the two species. This analysis identified 37 genes with *dN*/*dS* ratios greater than 1.1, indicating positive selective pressure for divergence (Supplementary Table 3).

**Figure 2:**
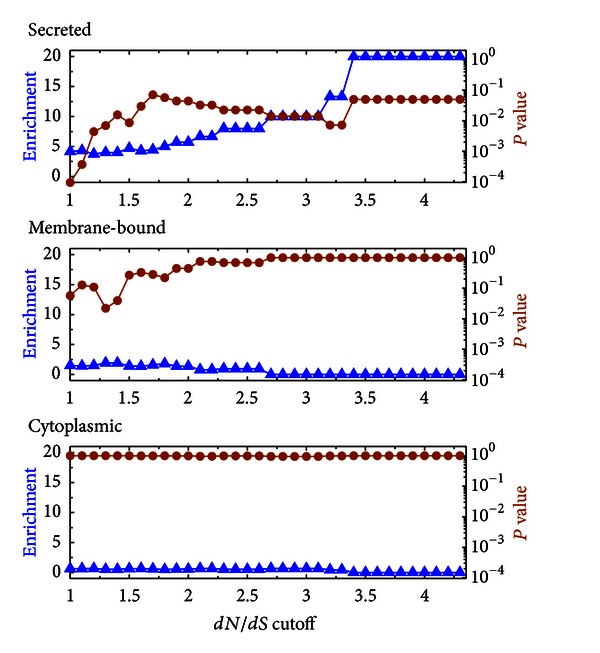
Protein localization and selective pressure. Association between protein localization and selective pressure. Abscissa: cutoffs used for the identification of positively selected proteins (are considered as positively selected proteins with *dN*/*dS* ratios higher than or equal to the cutoff). Ordinate: enrichment (triangles) and *P* value (circles) of the set of positively selected proteins for a given localization—either cytoplasmic, membrane-bound, or secreted.

Positively selected proteins have previously been shown to be associated with the surface of diverging pathogens, probably due to the necessity of the pathogen to alter its surface antigenicity to avoid the host immune response. [12] demonstrated this phenomenon in comparison of two related apicomplexan parasites, *Theileria annulata* and *T. parva*. Therefore, we used publicly available sequence analysis tools (see Section 4) to identify putative surface-associated or secreted proteins, that is, those with transmembrane domains, glycosylphosphatidylinositol anchors, signal sequences, or signal peptides (Supplementary Table 4). We used this information to evaluate the association between protein localization and the *dN*/*dS* ratio. Thus, we selected subsets of proteins having a high *dN*/*dS* ratio when considering different cutoff values. The enrichment of these subsets in proteins of given cellular localization was calculated as the fraction of proteins in a subset having a given localization divided by the fraction of proteins in the genome having this same localization. The statistical significance of this enrichment was evaluated by calculating a *P* value (the probability that this enrichment, or a better one, would be obtained by chance alone) using Fisher's exact test. The results (Figure 2) showed that positively selected proteins (*dN*/*dS* ratio equal or greater to 1.1) were significantly enriched in putatively secreted products; that is, 21.62% of the 37 positively selected proteins share this localization. This represents an enrichment of 4.16 and a *P* value of 9.48 × 10^−5^. A similar trend was observed for membrane-bound proteins (enrichment of 1.5, *P* value of 0.057). In contrast, typical cytoplasmic proteins showed strongly conserved *dN*/*dS* ratios; for example, glycolytic enzymes exhibit a mean *dN*/*dS* ratio of only 0.06, and enzymes of pyruvate metabolism exhibit a ratio of only 0.07 (Supplementary Table 4). These observations suggest that surface-associated and secreted proteins are strongly selected for divergence in *Cryptosporidium*.

We expected to observe a similar trend when examining the sequence identities of orthologous proteins in *C. hominis* and *C. parvum*; that is, surface-associated proteins should exhibit the greatest sequence divergence. Thus, we measured the identities of each protein orthologous pair in these two organisms and sorted them according to their cellular compartment. As it might be expected, the results of this analysis reinforced those of the *dN*/*dS* comparison. Thus, of the 145 protein pairs with identity lower than 90% (the average identity of all *C. hominis* and *C. parvum* genes being ~97%), 33 (22.76%) are predicted to be secreted proteins and 51 (35.17%) are predicted to be membrane-bound. This is 4.76-fold (*P* value of 8.21 × 10^−15^) and 1.8-fold (*P* value of 8.50 × 10^−6^) enrichment, respectively, over that expected by chance alone.

These results indicate that the two *Cryptosporidium* species have diverged most rapidly at the level of the proteins interacting with their environments, that is, the environment, the host, and the host immune system. This observation likely explains the significant host range and pathogenicity differences exhibited by the two pathogens.

## 3. Conclusions

Herein, we have compared the genomes of *C. hominis* and *C. parvum* to each other and to the genomes of related apicomplexan parasites. Our results show that despite the rather large sequence divergence of 3–5% between the two *Cryptosporidium* genomes, similar to that observed between humans and chimpanzees, the structure and composition of these two genomes are largely conserved. Not only is the number of chromosomes conserved at eight, but the two genomes are apparently completely collinear and each of the chromosomes in the two parasites is completely syntenic. Despite the synteny between *C. hominis* and *C. parvum*, almost no synteny was observed between the *Cryptosporidium* and *Plasmodium*. In contrast, gene composition of a chromosome fragments was highly conserved between these two genera, suggesting that the mechanisms by which *Cryptosporidium* evolved from its more genetically robust progenitors involved large scale gene deletion and more extensive *cis*- than *trans*-rearrangements. We also found no genes that are unique to either of the parasites, that is, present in only one of the two genomes. Comparison of the gene and pathway composition of the two parasites to those of other apicomplexans showed that the *Cryptosporidium* genome and metabolism are remarkably reduced, even more so than the *Theileria* spp. which have a smaller genome size. Finally, a comparison of the mutation rates of *Cryptosporidium* genes showed that those proteins associated with the surface of the parasite are being selected for rapid divergence, in stark contrast to the typical cytoplasmic proteins, which show a much higher level of conservation across the two species. This observation suggests that the phenotypic differences exhibited by *C. hominis* and *C. parvum* are due to selective forces exerted by the host parasite interaction largely upon the surface of the parasite.

## 4. Methods

### 4.1. Genome Comparison

Chromosome numbers, size, and sequences, as well as genome annotations (i.e., identification of the open reading frames) were obtained from the sequencing projects of *C. hominis* [9] and *C. parvum* [10]. In order to minimize the influence of annotation methodology in the accuracy of comparisons, *C. parvum* proteins were predicted *de novo* for this work, as previously described [9]. This was necessary since the versions of *C. parvum* proteins available in the general databases at the time of these analyses—GenBank and CryptoDB [17]—were missing around 400 proteins when compared to *C. hominis* or to our version of *C. parvum* gene predictions. Comparison of the organization of these two genomes was done first by obtaining restriction optical maps [18–26] for the SacI restriction enzyme, experimentally for *C. hominis* and *in silico* for *C. parvum*. The restriction fragment patterns generated by the optical mapping procedure were aligned for direct comparison using the Map Assembler algorithm [27–29]. For each given chromosome, discrepancies found in the alignment of the optical maps from the two species were investigated by the polymerase chain reaction (PCR). In brief, sequences ~3 kb upstream and downstream from the putative sites of discrepancy were retrieved from the *C. parvum* sequences. Primers directed towards the sites of discrepancy were designed and used in PCR reactions to assess and validate the presumed orientations of the contigs. Reactions were performed in volumes of 15 *μ*L containing 250 *μ*M dNTP, 2 ng/*μ*L of each oligonucleotide primer, 1.5 units of Taq DNA Polymerase (HotMaster, 5 Prime Gaithersburg, MD, USA), 15 ng of *C. parvum* (IA) DNA, and the appropriate buffer as provided by the manufacturer. The following PCR conditions were used: 98°C for 3 min, 35 cycles of 95°C for 30 sec, 49°C for 30 sec, and 68°C for 5 min. DNA amplification was assessed by agarose gel electrophoresis. Amplification products were taken as verification that the contigs from which the two primers in a reaction were selected were colocalized in the genome of *C. hominis*.

Genome annotations of the three plasmodia, *P. falciparum*, *P. knowlesi*, and *P. vivax*, were retrieved from the release 5.5 of the PlasmoDB database [30]. Orthologs with *C. parvum* were identified using OrthoMCL [31]. The enrichment of each chromosome of the three plasmodia genomes in orthologs identified in given chromosomes of *C. parvum* was statistically evaluated using Fisher's exact test. The ordering of the orthologs within chromosomes was evaluated for each pair of species by drawing dot plots.

### 4.2. Gene Complement Comparison

Gene content comparison was performed by identifying putative orthologs between the two parasites, as well as genes in each organism having no counterpart in the other one. Ortholog and paralog identification was performed using InParanoid [32], a tool that performed an automated bidirectional BLAST search [11] involving two proteomes. Genes found to be apparently unique in each of the two parasites were manually examined to identify and remove artifacts due to remaining gaps in the two sequences. Putative ortholog and paralog assignment involving all Apicomplexa were performed using OrthoMCL [31], and the results were further filtered and summarized by locally developed software tools.

### 4.3. Pathway Comparison

Comparison of the metabolic pathways in *C. hominis* and *C. parvum* was performed in two steps. First, the two genomes were annotated for the presence of the pathways defined in the KEGG [33] and BioCarta (http://www.biocarta.com/) databases by sequence similarity, as previously described [9]. To ensure results were completely comparable between the two species, we reannotated the proteins from *C. parvum* using the same methods employed for *C. hominis*. Second, the potential impairments of each pathway for each organism were determined by calculation of a completeness and a connectedness score [13, 14]. Each pathway putatively present in the query organism was compared to a canonical pathway that includes all the enzymatic reactions known to be present in any of the organisms available in KEGG for this pathway. Completeness is defined as the fraction of reactions present in the query organism when compared to the reactions of the canonical pathway. Connectedness is the fraction of intact paths between ports (input and output metabolites for a pathway) in the pathway according to the inferred set of reactions when compared to all the possible paths between the canonical ports in that pathway. Ports are defined as metabolites exchanged between the considered pathway and at least one other pathway; that is, a port is a metabolite that is used either as reactant or product in at least two pathways. To this list of ports, we added metabolites produced but never consumed by any known reaction, as well as metabolites consumed but never produced by any known reaction. These were considered artifacts due to either a missing reaction or the presence of a transporter. In both cases, these metabolites are eligible to be considered as ports for the pathways to which they belong. Finally, we defined the support score as the number of species in KEGG that contains information about the pathway being evaluated.

### 4.4. Selective Pressure Analysis

For the identification of genes under positive, negative, or neutral selective pressure, pairs of orthologs were aligned globally using the Smith-Waterman algorithm implemented in the FASTA package [34]. Nonsynonymous to synonymous substitution ratios (*dN*/*dS* ratio) were calculated using the Yang and Nielson method [35] as implemented in the PAML package [36]. A software pipeline, MUTATION HUNTER, available at http://github.com/ajmazurie/MutationsHunter, was written to automate this task. Automated annotation of the protein sequences was performed on *C. hominis* sequences to predict the presence of transmembrane domains using tmhmm 2.0 [37], glycosylphosphatidylinositol anchors using GPI-SOM [38], and signal sequences and peptides using SIGNALP 3.0 [39].

## Supplementary Material

Supplementary Figure 1: Synteny of *Plasmodia* and *Cryptosporidia* genus Position of the orthologs identified in the genomes of *C. parvum*, *P. falciparum*, *P. knowlesi* and *P. vivax*. For each pair of species, a green cross denotes an ortholog present in the same strand in both genomes, while a red cross denotes an inversion.
Supplementary Figure 2: Genome Compactness Comparison of the genome compactness of well-studied apicomplexans, as the average number of putative paralogs per genes.
Supplementary Figure 3: Pathway Scores See Figure 1.
Supplementary Table 1: Chromosomes Composition Results of the statistical evaluation performed using the Fisher's exact test of the enrichment of the *Plasmodia* chromosomes in orthologs identified in *C. parvum*. Values of enrichment are given as the log2 of the ratio between the fraction of orthologs in a given *Plasmodium* chromosome coming from a same chromosome of *C. parvum*, and the fraction of orthologs in the whole *Plasmodium* genome coming from this *C. parvum* chromosome. A value of 0 (ratio of 1) means that the *Plasmodium* chromosome contains proportionally as many orthologs from the *C. parvum* chromosome considered as in the whole genome. The values obtained show that the *Plasmodium* chromosomes significantly retain the composition of the *C. parvum* chromosomes. P-values below 0.05 were discarded.
Supplementary Table 2: Pathway Scores Completeness, connectedness and support scores of the inferred metabolic pathways of *C. hominis* and *C. parvum*, along with nine other apicomplexans and an external reference, *S. cerevisiae* (see text).
Supplementary Table 3: Orthologs Sequence Comparison Results of the pairs of putative orthologs sequences comparisons. Identity: percentage of identity between the two protein sequences. dN/dS: non-synonymous on synonymous substitution rate ratio, or None if undefined (when dS equals to zero). Ti/Tv: transition on transversion ratio.
Supplementary Table 4: Protein Localizations Predicted protein localization, based on the putative presence of transmembrane domains, GPI anchor and/or signal peptides (see text). For each pair of orthologs, the electronic annotation was done on the *C. hominis* sequence (identifier in the first column). *P*(signal peptide) and *P*(signal anchor): probability of a signal peptide and signal anchor as calculated by SignalP. # GPI anchors: putative number of GPI anchors as predicted by gpi-som. # TM domains: putative number of transmembrane helices as predicted by tmhmm.
