# Supplementary material for: Comparative Genomics of Cryptosporidium
Source: Int J Genomics. 2013 May 2;2013:832756. doi: 10.1155/2013/832756 (PMC3659464; doi:10.1155/2013/832756)
Supplement: Supplementary file 1 — Supplementary Figure 1: Synteny of Plasmodia and Cryptosporidia genus Position of the orthologs identified in the genomes of C. parvum, P. falciparum, P. knowlesi and P. vivax. For each pair of species, a green cross denotes an ortholog present in the same strand in both genomes, while a red cross denotes an inversion. Supplementary Figure 2: Genome Compactness Comparison of the genome compactness of well-studied apicomplexans, as the average number of putative paralogs per genes. Supplementary Figure 3: Pathway Scores See Figure 1. Supplementary Table 1: Chromosomes Composition Results of the statistical evaluation performed using the Fisher's exact test of the enrichment of the Plasmodia chromosomes in orthologs identified in C. parvum. Values of enrichment are given as the log2 of the ratio between the fraction of orthologs in a given Plasmodium chromosome coming from a same chromosome of C. parvum, and the fraction of orthologs in the whole Plasmodium genome coming from this C. parvum chromosome. A value of 0 (ratio of 1) means that the Plasmodium chromosome contains proportionally as many orthologs from the C. parvum chromosome considered as in the whole genome. The values obtained show that the Plasmodium chromosomes significantly retain the composition of the C. parvum chromosomes. P-values below 0.05 were discarded. Supplementary Table 2: Pathway Scores Completeness, connectedness and support scores of the inferred metabolic pathways of C. hominis and C. parvum, along with nine other apicomplexans and an external reference, S. cerevisiae (see text). Supplementary Table 3: Orthologs Sequence Comparison Results of the pairs of putative orthologs sequences comparisons. Identity: percentage of identity between the two protein sequences. dN/dS: non-synonymous on synonymous substitution rate ratio, or None if undefined (when dS equals to zero). Ti/Tv: transition on transversion ratio. Supplementary Table 4: Protein Localizations Predicted protein localization, bas [file 832756.f1.zip › supplementary_figure_3-pathway_scores.pdf]

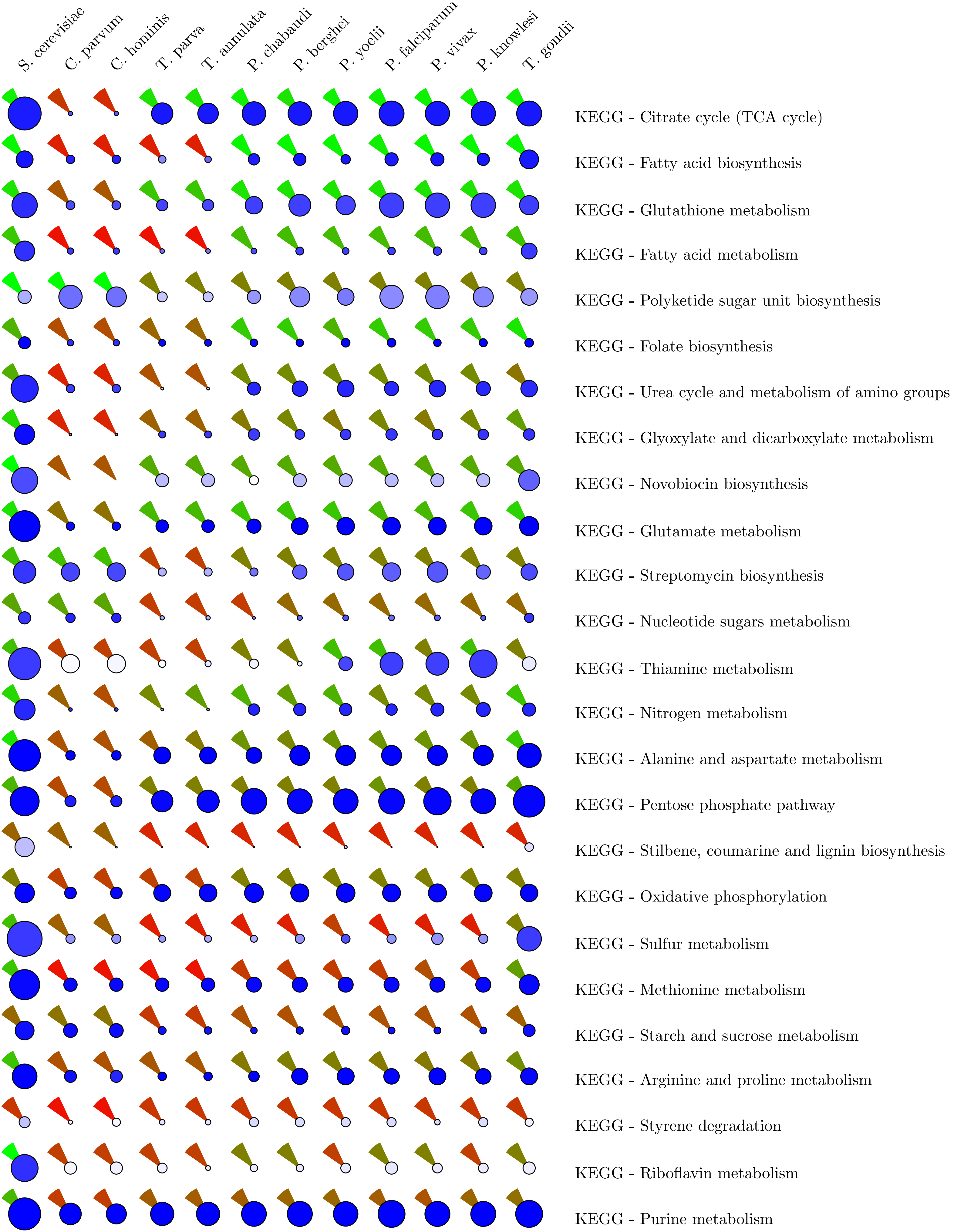
S. cerevisiae
C. parvum
C. hominis
T. parva
T. annulata
P. chabaudi
P. berghei
P. yoelii
P. falciparum
P. vivax
P. knowlesi
T. gondii
KEGG - Citrate cycle (TCA cycle)
KEGG - Fatty acid biosynthesis
KEGG - Glutathione metabolism
KEGG - Fatty acid metabolism
KEGG - Polyketide sugar unit biosynthesis
KEGG - Folate biosynthesis
KEGG - Urea cycle and metabolism of amino groups
KEGG - Glyoxylate and dicarboxylate metabolism
KEGG - Novobiocin biosynthesis
KEGG - Glutamate metabolism
KEGG - Streptomycin biosynthesis
KEGG - Nucleotide sugars metabolism
KEGG - Thiamine metabolism
KEGG - Nitrogen metabolism
KEGG - Alanine and aspartate metabolism
KEGG - Pentose phosphate pathway
KEGG - Stilbene, coumarine and lignin biosynthesis
KEGG - Oxidative phosphorylation
KEGG - Sulfur metabolism
KEGG - Methionine metabolism
KEGG - Starch and sucrose metabolism
KEGG - Arginine and proline metabolism
KEGG - Styrene degradation
KEGG - Riboflavin metabolism
KEGG - Purine metabolism

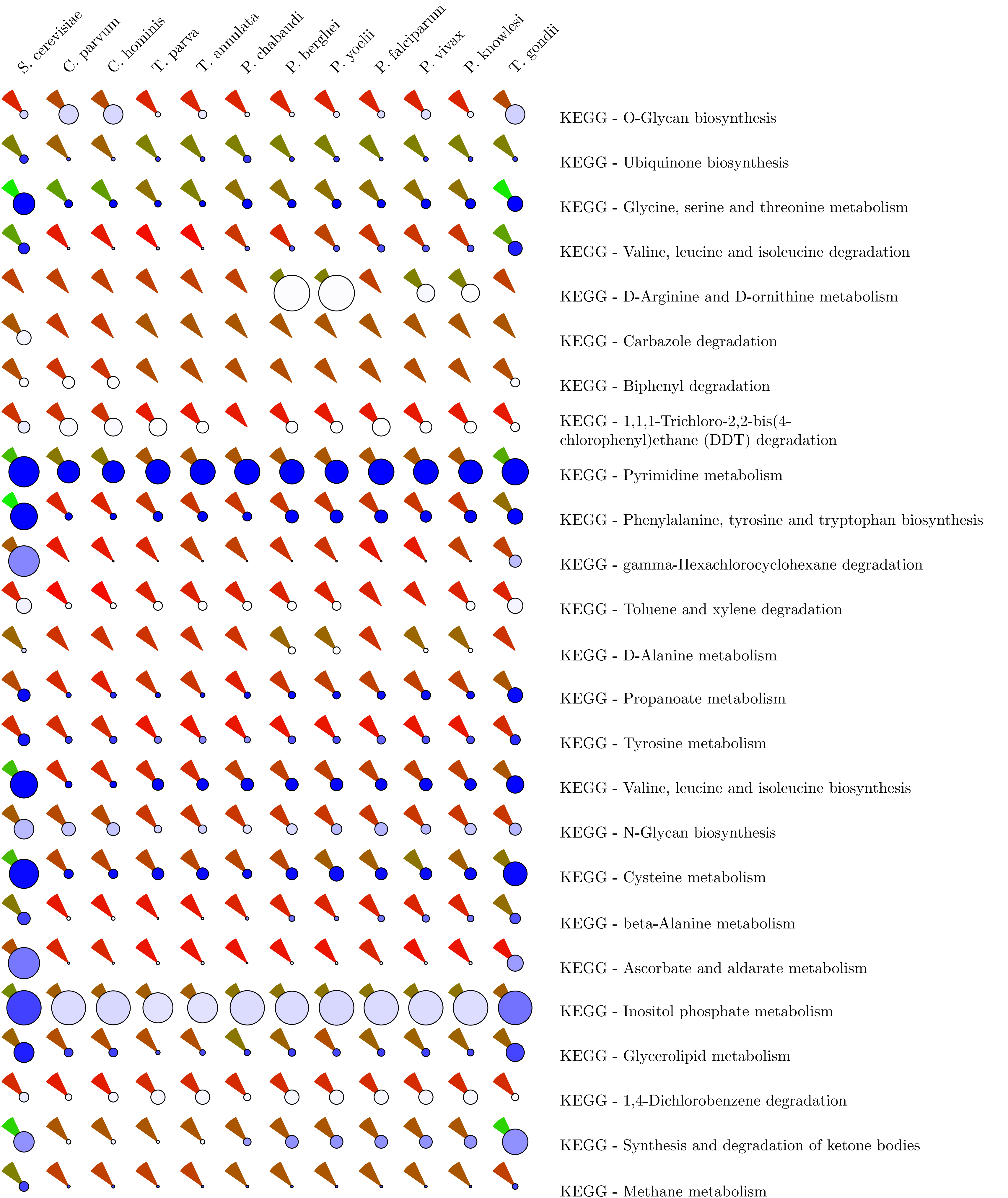
S. cerevisiae
C. parvum
C. hominis
T. parva
T. annulata
P. chabaudi
P. berghei
P. yoelii
P. falciparum
P. vivax
P. knowlesi
T. gondii
KEGG - O-Glycan biosynthesis
KEGG - Ubiquinone biosynthesis
KEGG - Glycine, serine and threonine metabolism
KEGG - Valine, leucine and isoleucine degradation
KEGG - D-Arginine and D-ornithine metabolism
KEGG - Carbazole degradation
KEGG - Biphenyl degradation
KEGG - 1,1,1-Trichloro-2,2-bis(4-chlorophenyl)ethane (DDT) degradation
KEGG - Pyrimidine metabolism
KEGG - Phenylalanine, tyrosine and tryptophan biosynthesis
KEGG - gamma-Hexachlorocyclohexane degradation
KEGG - Toluene and xylene degradation
KEGG - D-Alanine metabolism
KEGG - Propanoate metabolism
KEGG - Tyrosine metabolism
KEGG - Valine, leucine and isoleucine biosynthesis
KEGG - N-Glycan biosynthesis
KEGG - Cysteine metabolism
KEGG - beta-Alanine metabolism
KEGG - Ascorbate and aldarate metabolism
KEGG - Inositol phosphate metabolism
KEGG - Glycerolipid metabolism
KEGG - 1,4-Dichlorobenzene degradation
KEGG - Synthesis and degradation of ketone bodies
KEGG - Methane metabolism

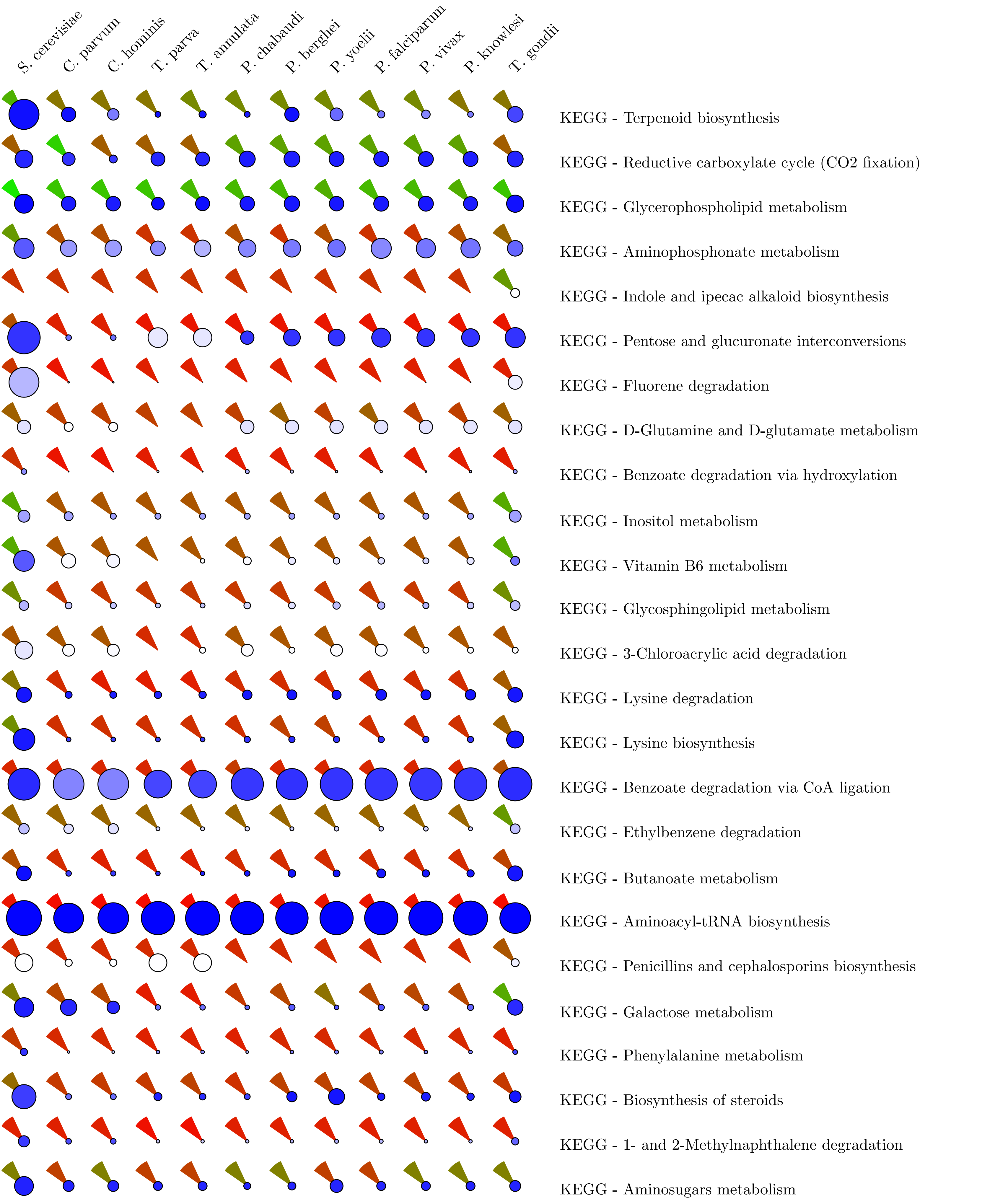
S. cerevisiae
C. parvum
C. hominis
T. parva
T. annulata
P. chabaudi
P. berghei
P. yoelii
P. falciparum
P. vivax
P. knowlesi
T. gondii
KEGG - Terpenoid biosynthesis
KEGG - Reductive carboxylate cycle (CO2 fixation)
KEGG - Glycerophospholipid metabolism
KEGG - Aminophosphonate metabolism
KEGG - Indole and ipecac alkaloid biosynthesis
KEGG - Pentose and glucuronate interconversions
KEGG - Fluorene degradation
KEGG - D-Glutamine and D-glutamate metabolism
KEGG - Benzoate degradation via hydroxylation
KEGG - Inositol metabolism
KEGG - Vitamin B6 metabolism
KEGG - Glycosphingolipid metabolism
KEGG - 3-Chloroacrylic acid degradation
KEGG - Lysine degradation
KEGG - Lysine biosynthesis
KEGG - Benzoate degradation via CoA ligation
KEGG - Ethylbenzene degradation
KEGG - Butanoate metabolism
KEGG - Aminoacyl-tRNA biosynthesis
KEGG - Penicillins and cephalosporins biosynthesis
KEGG - Galactose metabolism
KEGG - Phenylalanine metabolism
KEGG - Biosynthesis of steroids
KEGG - 1- and 2-Methylnaphthalene degradation
KEGG - Aminosugars metabolism

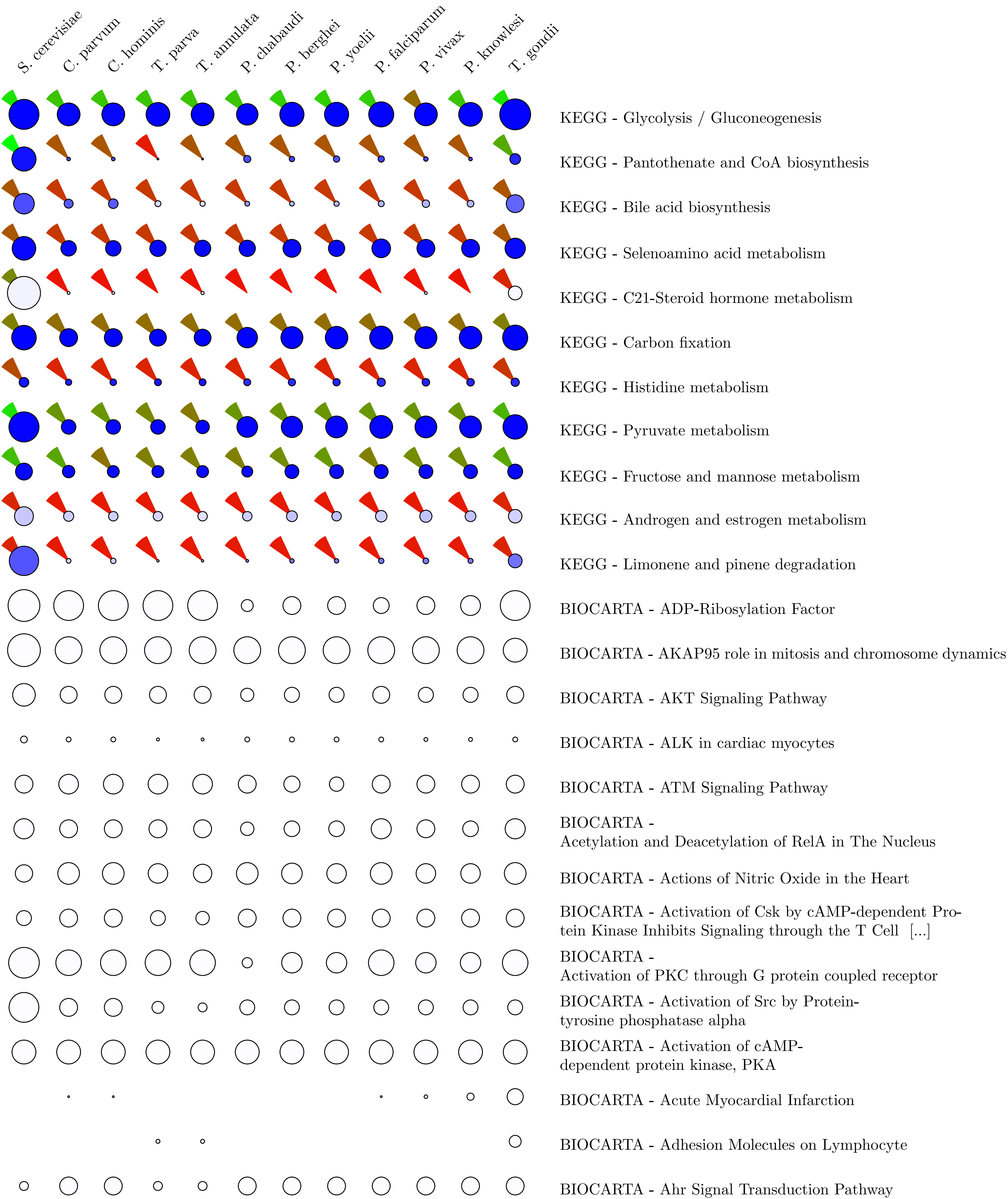
S. cerevisiae
C. parvum
C. hominis
T. parva
T. annulata
P. chabaudi
P. berghei
P. yoelii
P. falciparum
P. vivax
P. knowlesi
T. gondii
KEGG - Glycolysis / Gluconeogenesis
KEGG - Pantothenate and CoA biosynthesis
KEGG - Bile acid biosynthesis
KEGG - Selenoamino acid metabolism
KEGG - C21-Steroid hormone metabolism
KEGG - Carbon fixation
KEGG - Histidine metabolism
KEGG - Pyruvate metabolism
KEGG - Fructose and mannose metabolism
KEGG - Androgen and estrogen metabolism
KEGG - Limonene and pinene degradation
BIOCARTA - ADP-Ribosylation Factor
BIOCARTA - AKAP95 role in mitosis and chromosome dynamics
BIOCARTA - AKT Signaling Pathway
BIOCARTA - ALK in cardiac myocytes
BIOCARTA - ATM Signaling Pathway
BIOCARTA - Acetylation and Deacetylation of RelA in The Nucleus
BIOCARTA - Actions of Nitric Oxide in the Heart
BIOCARTA - Activation of Csk by cAMP-dependent Protein Kinase Inhibits Signaling through the T Cell [...]
BIOCARTA - Activation of PKC through G protein coupled receptor
BIOCARTA - Activation of Src by Protein-tyrosine phosphatase alpha
BIOCARTA - Activation of cAMP-dependent protein kinase, PKA
BIOCARTA - Acute Myocardial Infarction
BIOCARTA - Adhesion Molecules on Lymphocyte
BIOCARTA - Ahr Signal Transduction Pathway

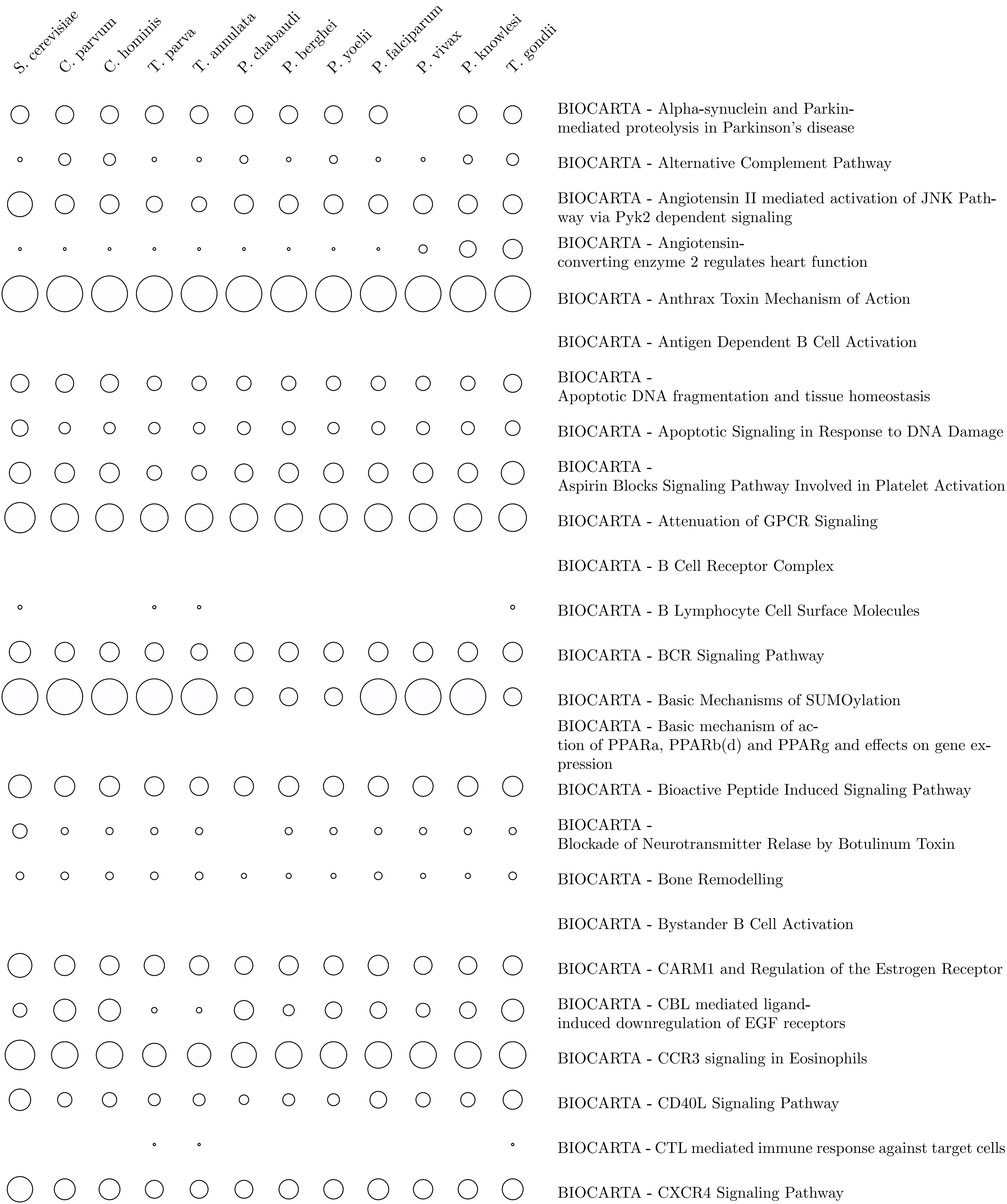
S. cerevisiae
C. parvum
C. hominis
T. parva
T. annulata
P. chabaudi
P. berghei
P. yoelii
P. falciparum
P. vivax
P. knowlesi
T. gondii
BIOCARTA - Alpha-synuclein and Parkin-mediated proteolysis in Parkinson's disease
BIOCARTA - Alternative Complement Pathway
BIOCARTA - Angiotensin II mediated activation of JNK Pathway via Pyk2 dependent signaling
BIOCARTA - Angiotensin-converting enzyme 2 regulates heart function
BIOCARTA - Anthrax Toxin Mechanism of Action
BIOCARTA - Antigen Dependent B Cell Activation
BIOCARTA - Apoptotic DNA fragmentation and tissue homeostasis
BIOCARTA - Apoptotic Signaling in Response to DNA Damage
BIOCARTA - Aspirin Blocks Signaling Pathway Involved in Platelet Activation
BIOCARTA - Attenuation of GPCR Signaling
BIOCARTA - B Cell Receptor Complex
BIOCARTA - B Lymphocyte Cell Surface Molecules
BIOCARTA - BCR Signaling Pathway
BIOCARTA - Basic Mechanisms of SUMOylation
BIOCARTA - Basic mechanism of action of PPARa, PPARb(d) and PPARg and effects on gene expression
BIOCARTA - Bioactive Peptide Induced Signaling Pathway
BIOCARTA - Blockade of Neurotransmitter Relase by Botulinum Toxin
BIOCARTA - Bone Remodelling
BIOCARTA - Bystander B Cell Activation
BIOCARTA - CARM1 and Regulation of the Estrogen Receptor
BIOCARTA - CBL mediated ligand-induced downregulation of EGF receptors
BIOCARTA - CCR3 signaling in Eosinophils
BIOCARTA - CD40L Signaling Pathway
BIOCARTA - CTL mediated immune response against target cells
BIOCARTA - CXCR4 Signaling Pathway

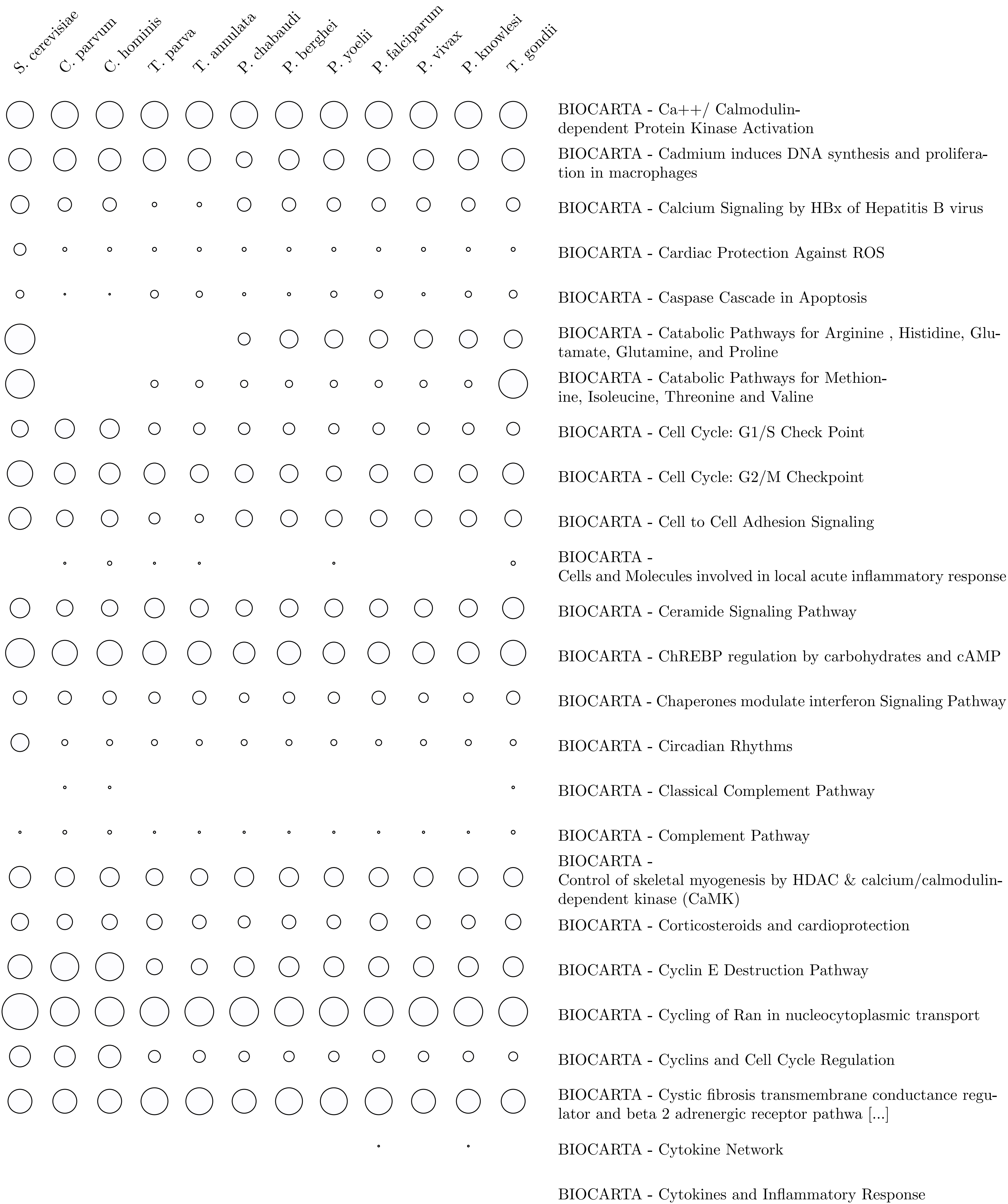
S. cerevisiae
C. parvum
C. hominis
T. parva
T. annulata
P. chabaudi
P. berghei
P. yoelii
P. falciparum
P. vivax
P. knowlesi
T. gondii
BIOCARTA - Ca++/ Calmodulin-dependent Protein Kinase Activation
BIOCARTA - Cadmium induces DNA synthesis and proliferation in macrophages
BIOCARTA - Calcium Signaling by HBx of Hepatitis B virus
BIOCARTA - Cardiac Protection Against ROS
BIOCARTA - Caspase Cascade in Apoptosis
BIOCARTA - Catabolic Pathways for Arginine , Histidine, Glutamate, Glutamine, and Proline
BIOCARTA - Catabolic Pathways for Methionine, Isoleucine, Threonine and Valine
BIOCARTA - Cell Cycle: G1/S Check Point
BIOCARTA - Cell Cycle: G2/M Checkpoint
BIOCARTA - Cell to Cell Adhesion Signaling
BIOCARTA - Cells and Molecules involved in local acute inflammatory response
BIOCARTA - Ceramide Signaling Pathway
BIOCARTA - ChREBP regulation by carbohydrates and cAMP
BIOCARTA - Chaperones modulate interferon Signaling Pathway
BIOCARTA - Circadian Rhythms
BIOCARTA - Classical Complement Pathway
BIOCARTA - Complement Pathway
BIOCARTA - Control of skeletal myogenesis by HDAC & calcium/calmodulin-dependent kinase (CaMK)
BIOCARTA - Corticosteroids and cardioprotection
BIOCARTA - Cyclin E Destruction Pathway
BIOCARTA - Cycling of Ran in nucleocytoplasmic transport
BIOCARTA - Cyclins and Cell Cycle Regulation
BIOCARTA - Cystic fibrosis transmembrane conductance regulator and beta 2 adrenergic receptor pathwa [...]
BIOCARTA - Cytokine Network
BIOCARTA - Cytokines and Inflammatory Response

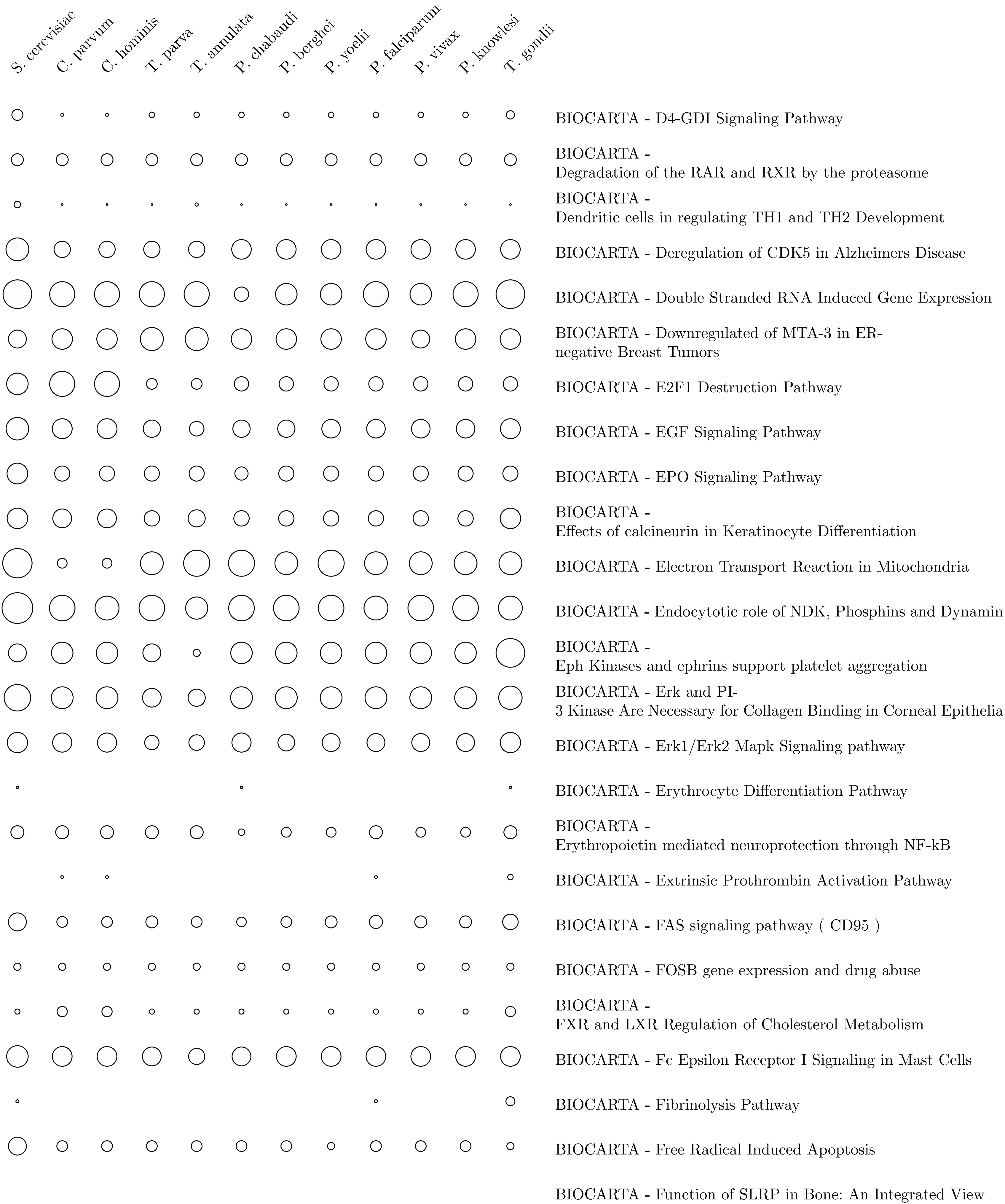
S. cerevisiae
C. parvum
C. hominis
T. parva
T. annulata
P. chabaudi
P. berghei
P. yoelii
P. falciparum
P. vivax
P. knowlesi
T. gondii
BIOCARTA - D4-GDI Signaling Pathway
BIOCARTA - Degradation of the RAR and RXR by the proteasome
BIOCARTA - Dendritic cells in regulating TH1 and TH2 Development
BIOCARTA - Deregulation of CDK5 in Alzheimers Disease
BIOCARTA - Double Stranded RNA Induced Gene Expression
BIOCARTA - Downregulated of MTA-3 in ER-negative Breast Tumors
BIOCARTA - E2F1 Destruction Pathway
BIOCARTA - EGF Signaling Pathway
BIOCARTA - EPO Signaling Pathway
BIOCARTA - Effects of calcineurin in Keratinocyte Differentiation
BIOCARTA - Electron Transport Reaction in Mitochondria
BIOCARTA - Endocytotic role of NDK, Phosphins and Dynamin
BIOCARTA - Eph Kinases and ephrins support platelet aggregation
BIOCARTA - Erk and PI-3 Kinase Are Necessary for Collagen Binding in Corneal Epithelia
BIOCARTA - Erk1/Erk2 Mapk Signaling pathway
BIOCARTA - Erythrocyte Differentiation Pathway
BIOCARTA - Erythropoietin mediated neuroprotection through NF-kB
BIOCARTA - Extrinsic Prothrombin Activation Pathway
BIOCARTA - FAS signaling pathway ( CD95 )
BIOCARTA - FOSB gene expression and drug abuse
BIOCARTA - FXR and LXR Regulation of Cholesterol Metabolism
BIOCARTA - Fc Epsilon Receptor I Signaling in Mast Cells
BIOCARTA - Fibrinolysis Pathway
BIOCARTA - Free Radical Induced Apoptosis
BIOCARTA - Function of SLRP in Bone: An Integrated View

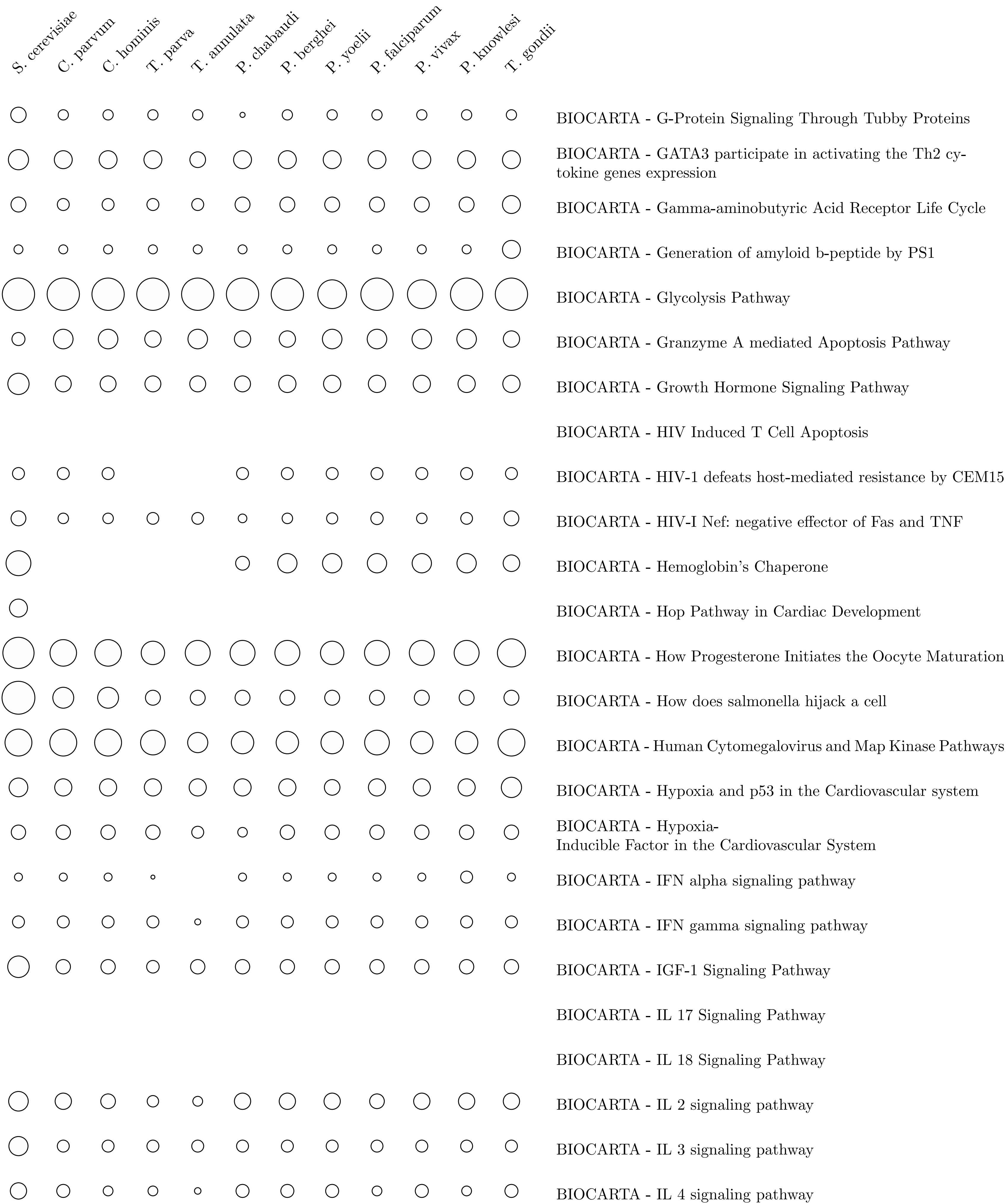
S. cerevisiae
C. parvum
C. hominis
T. parva
T. annulata
P. chabaudi
P. berghei
P. yoelii
P. falciparum
P. vivax
P. knowlesi
T. gondii
BIOCARTA - G-Protein Signaling Through Tubby Proteins
BIOCARTA - GATA3 participate in activating the Th2 cytokine genes expression
BIOCARTA - Gamma-aminobutyric Acid Receptor Life Cycle
BIOCARTA - Generation of amyloid b-peptide by PS1
BIOCARTA - Glycolysis Pathway
BIOCARTA - Granzyme A mediated Apoptosis Pathway
BIOCARTA - Growth Hormone Signaling Pathway
BIOCARTA - HIV Induced T Cell Apoptosis
BIOCARTA - HIV-1 defeats host-mediated resistance by CEM15
BIOCARTA - HIV-I Nef: negative effector of Fas and TNF
BIOCARTA - Hemoglobin's Chaperone
BIOCARTA - Hop Pathway in Cardiac Development
BIOCARTA - How Progesterone Initiates the Oocyte Maturation
BIOCARTA - How does salmonella hijack a cell
BIOCARTA - Human Cytomegalovirus and Map Kinase Pathways
BIOCARTA - Hypoxia and p53 in the Cardiovascular system
BIOCARTA - Hypoxia-Inducible Factor in the Cardiovascular System
BIOCARTA - IFN alpha signaling pathway
BIOCARTA - IFN gamma signaling pathway
BIOCARTA - IGF-1 Signaling Pathway
BIOCARTA - IL 17 Signaling Pathway
BIOCARTA - IL 18 Signaling Pathway
BIOCARTA - IL 2 signaling pathway
BIOCARTA - IL 3 signaling pathway
BIOCARTA - IL 4 signaling pathway

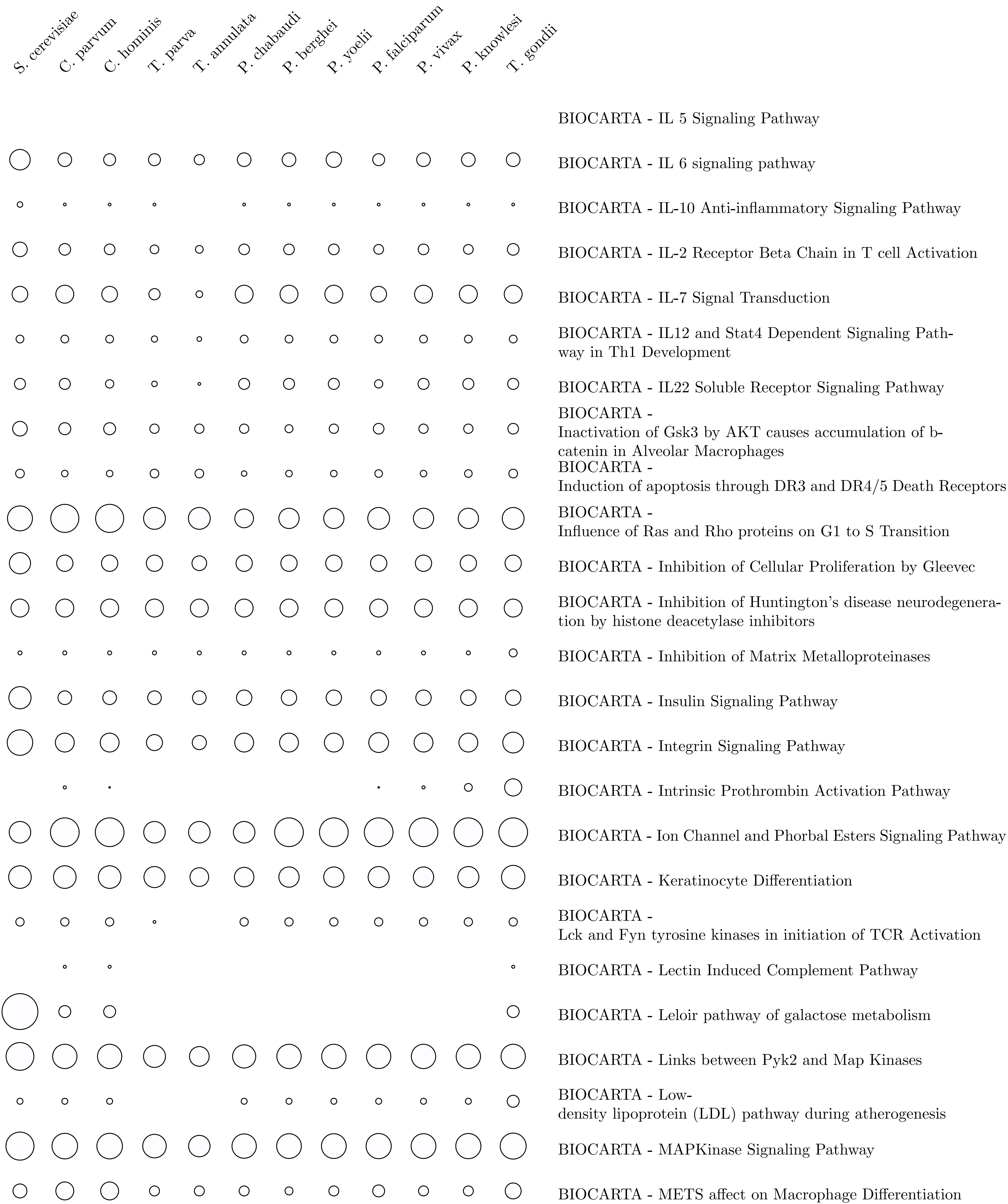
S. cerevisiae
C. parvum
C. hominis
T. parva
T. annulata
P. chabaudi
P. berghei
P. yoelii
P. falciparum
P. vivax
P. knowlesi
T. gondii
BIOCARTA - IL 5 Signaling Pathway
BIOCARTA - IL 6 signaling pathway
BIOCARTA - IL-10 Anti-inflammatory Signaling Pathway
BIOCARTA - IL-2 Receptor Beta Chain in T cell Activation
BIOCARTA - IL-7 Signal Transduction
BIOCARTA - IL12 and Stat4 Dependent Signaling Pathway in Th1 Development
BIOCARTA - IL22 Soluble Receptor Signaling Pathway
BIOCARTA - Inactivation of Gsk3 by AKT causes accumulation of b-catenin in Alveolar Macrophages
BIOCARTA - Induction of apoptosis through DR3 and DR4/5 Death Receptors
BIOCARTA - Influence of Ras and Rho proteins on G1 to S Transition
BIOCARTA - Inhibition of Cellular Proliferation by Gleevec
BIOCARTA - Inhibition of Huntington's disease neurodegeneration by histone deacetylase inhibitors
BIOCARTA - Inhibition of Matrix Metalloproteinases
BIOCARTA - Insulin Signaling Pathway
BIOCARTA - Integrin Signaling Pathway
BIOCARTA - Intrinsic Prothrombin Activation Pathway
BIOCARTA - Ion Channel and Phorbal Esters Signaling Pathway
BIOCARTA - Keratinocyte Differentiation
BIOCARTA - Lck and Fyn tyrosine kinases in initiation of TCR Activation
BIOCARTA - Lectin Induced Complement Pathway
BIOCARTA - Leloir pathway of galactose metabolism
BIOCARTA - Links between Pyk2 and Map Kinases
BIOCARTA - Low-density lipoprotein (LDL) pathway during atherogenesis
BIOCARTA - MAPKinase Signaling Pathway
BIOCARTA - METS affect on Macrophage Differentiation

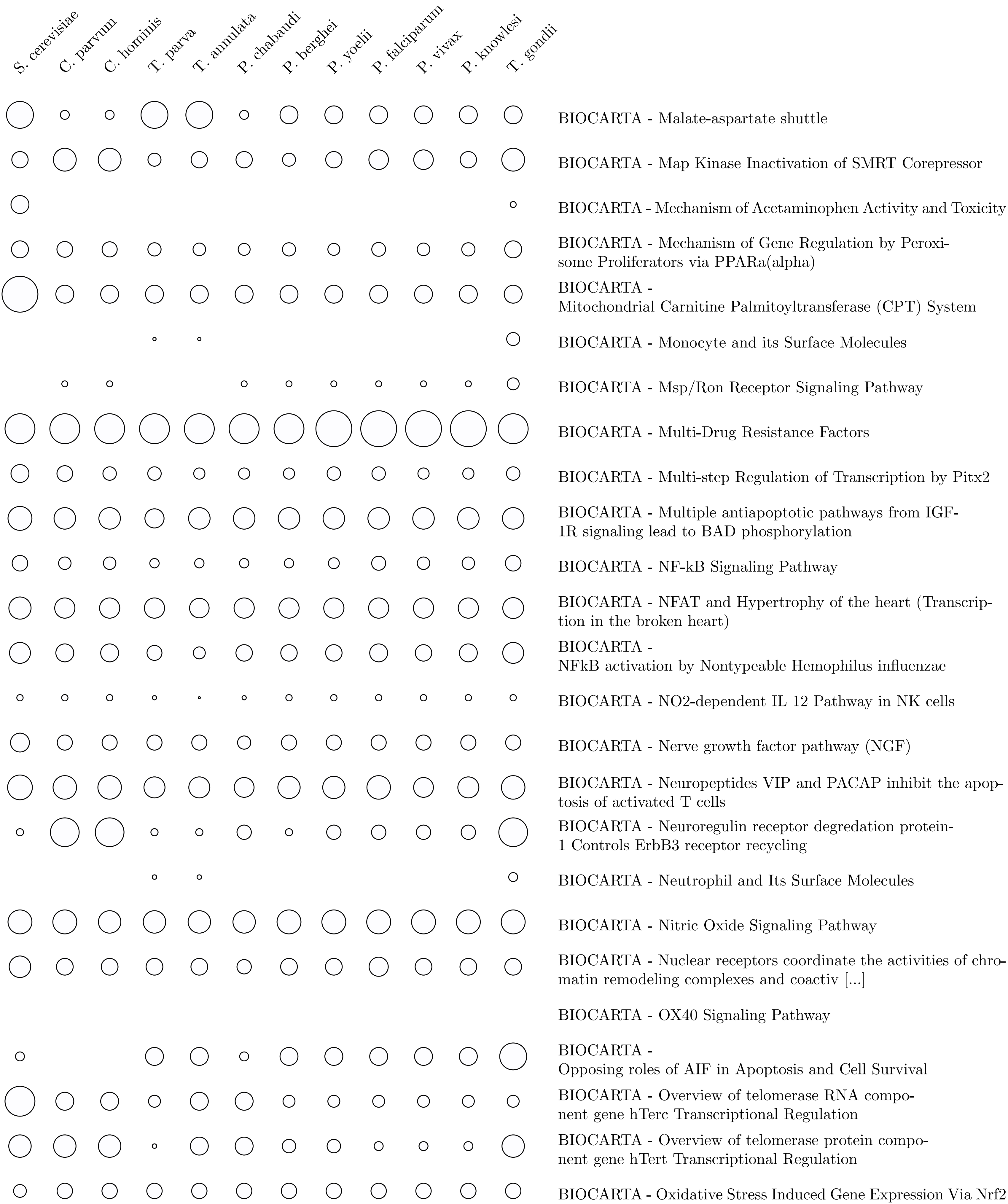
S. cerevisiae
C. parvum
C. hominis
T. parva
T. annulata
P. chabaudi
P. berghei
P. yoelii
P. falciparum
P. vivax
P. knowlesi
T. gondii
BIOCARTA - Malate-aspartate shuttle
BIOCARTA - Map Kinase Inactivation of SMRT Corepressor
BIOCARTA - Mechanism of Acetaminophen Activity and Toxicity
BIOCARTA - Mechanism of Gene Regulation by Peroxisome Proliferators via PPARa(alpha)
BIOCARTA - Mitochondrial Carnitine Palmitoyltransferase (CPT) System
BIOCARTA - Monocyte and its Surface Molecules
BIOCARTA - Msp/Ron Receptor Signaling Pathway
BIOCARTA - Multi-Drug Resistance Factors
BIOCARTA - Multi-step Regulation of Transcription by Pitx2
BIOCARTA - Multiple antiapoptotic pathways from IGF-1R signaling lead to BAD phosphorylation
BIOCARTA - NF-kB Signaling Pathway
BIOCARTA - NFAT and Hypertrophy of the heart (Transcription in the broken heart)
BIOCARTA - NFkB activation by Nontypeable Hemophilus influenzae
BIOCARTA - NO2-dependent IL 12 Pathway in NK cells
BIOCARTA - Nerve growth factor pathway (NGF)
BIOCARTA - Neuropeptides VIP and PACAP inhibit the apoptosis of activated T cells
BIOCARTA - Neuroregulin receptor degredation protein-1 Controls ErbB3 receptor recycling
BIOCARTA - Neutrophil and Its Surface Molecules
BIOCARTA - Nitric Oxide Signaling Pathway
BIOCARTA - Nuclear receptors coordinate the activities of chromatin remodeling complexes and coactiv [...]
BIOCARTA - OX40 Signaling Pathway
BIOCARTA - Opposing roles of AIF in Apoptosis and Cell Survival
BIOCARTA - Overview of telomerase RNA component gene hTerc Transcriptional Regulation
BIOCARTA - Overview of telomerase protein component gene hTert Transcriptional Regulation
BIOCARTA - Oxidative Stress Induced Gene Expression Via Nrf2

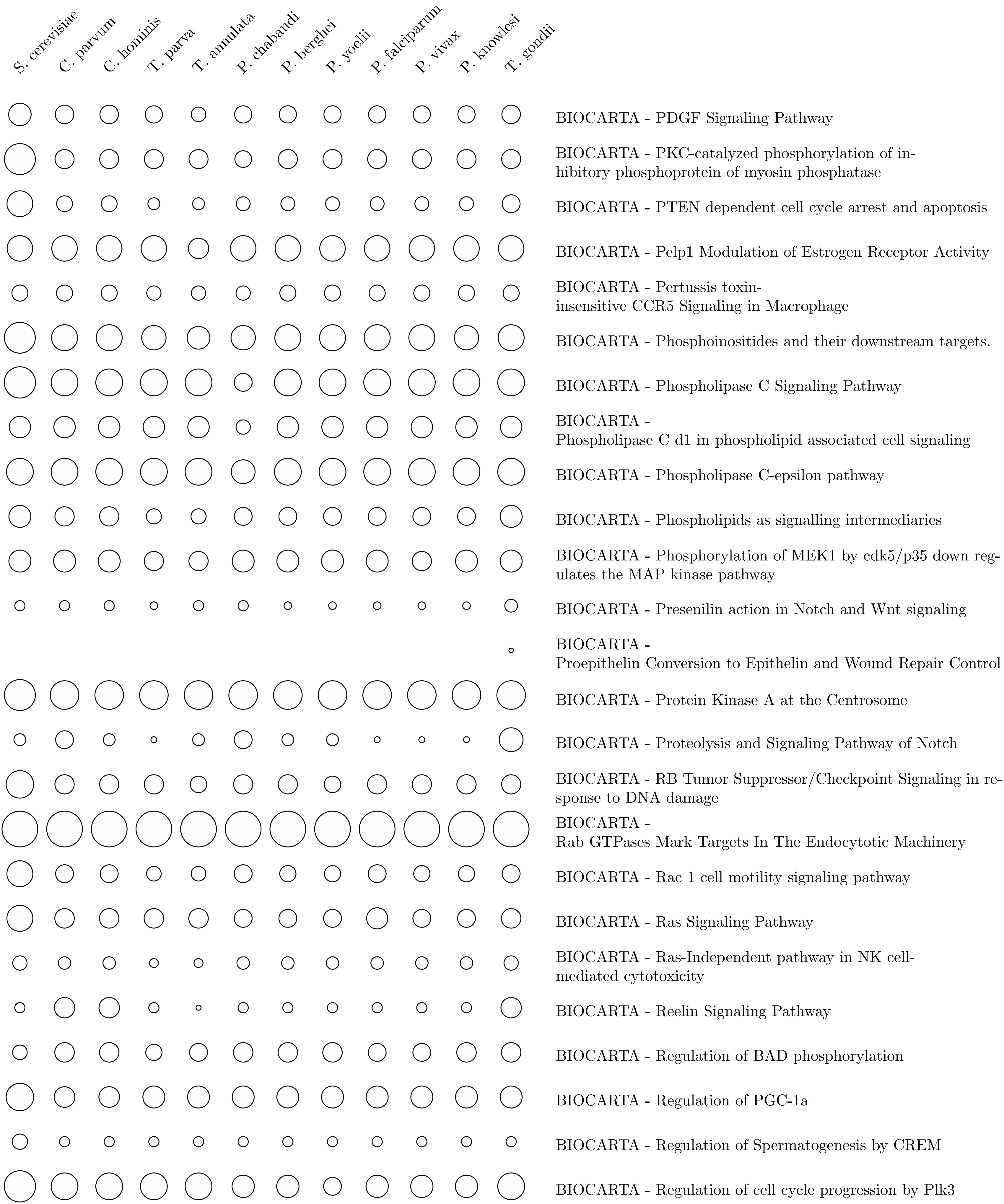

S. cerevisiae
C. parvum
C. hominis
T. parva
T. annulata
P. chabaudi
P. berghei
P. yoelii
P. falciparum
P. vivax
P. knowlesi
T. gondii
BIOCARTA - PDGF Signaling Pathway
BIOCARTA - PKC-catalyzed phosphorylation of inhibitory phosphoprotein of myosin phosphatase
BIOCARTA - PTEN dependent cell cycle arrest and apoptosis
BIOCARTA - Pelp1 Modulation of Estrogen Receptor Activity
BIOCARTA - Pertussis toxin-insensitive CCR5 Signaling in Macrophage
BIOCARTA - Phosphoinositides and their downstream targets.
BIOCARTA - Phospholipase C Signaling Pathway
BIOCARTA - Phospholipase C d1 in phospholipid associated cell signaling
BIOCARTA - Phospholipase C-epsilon pathway
BIOCARTA - Phospholipids as signalling intermediaries
BIOCARTA - Phosphorylation of MEK1 by cdk5/p35 down regulates the MAP kinase pathway
BIOCARTA - Presenilin action in Notch and Wnt signaling
BIOCARTA - Proepithelin Conversion to Epithelin and Wound Repair Control
BIOCARTA - Protein Kinase A at the Centrosome
BIOCARTA - Proteolysis and Signaling Pathway of Notch
BIOCARTA - RB Tumor Suppressor/Checkpoint Signaling in response to DNA damage
BIOCARTA - Rab GTPases Mark Targets In The Endocytotic Machinery
BIOCARTA - Rac 1 cell motility signaling pathway
BIOCARTA - Ras Signaling Pathway
BIOCARTA - Ras-Independent pathway in NK cell-mediated cytotoxicity
BIOCARTA - Reelin Signaling Pathway
BIOCARTA - Regulation of BAD phosphorylation
BIOCARTA - Regulation of PGC-1a
BIOCARTA - Regulation of Spermatogenesis by CREM
BIOCARTA - Regulation of cell cycle progression by Plk3

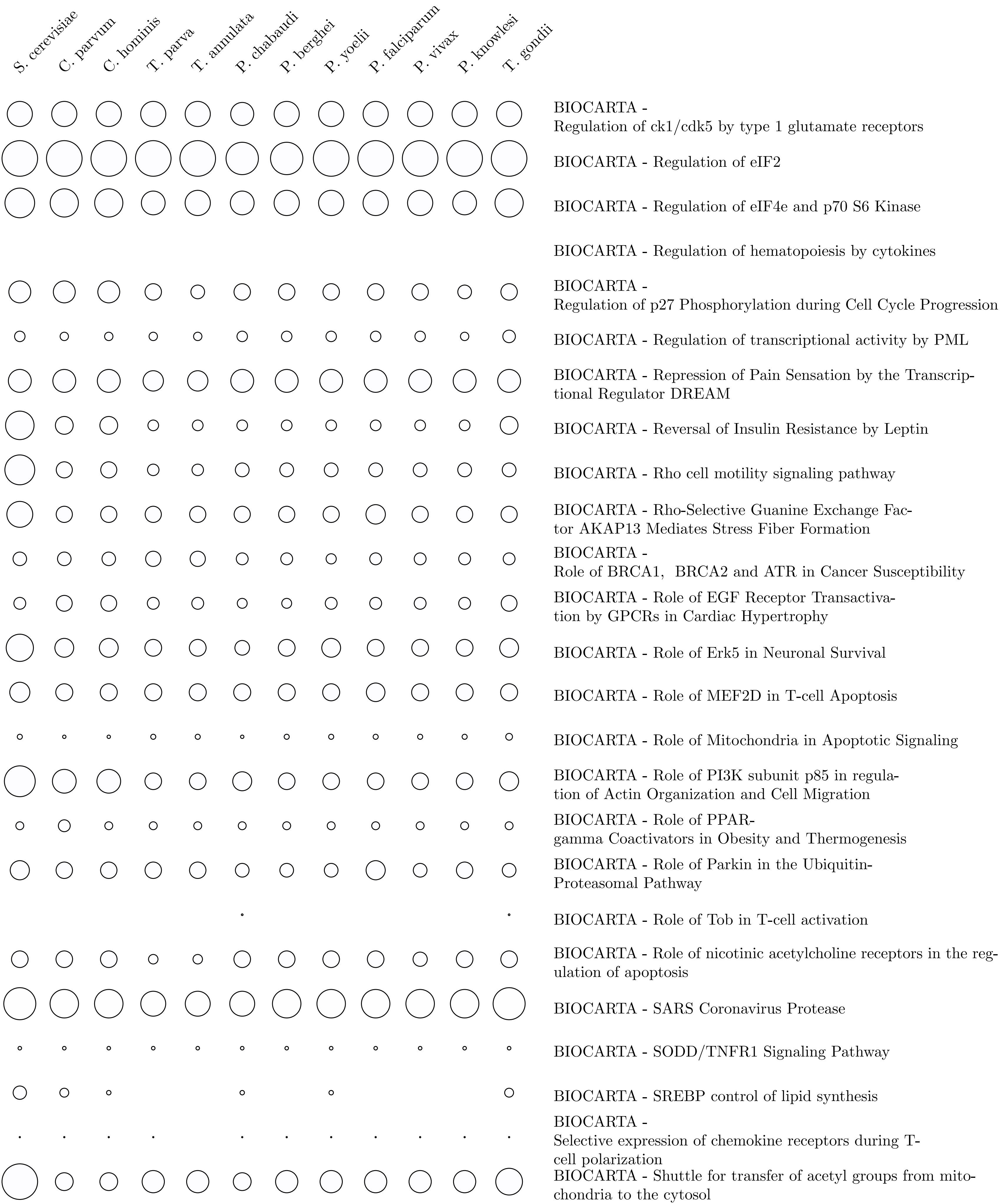
S. cerevisiae
C. parvum
C. hominis
T. parva
T. annulata
P. chabaudi
P. berghei
P. yoelii
P. falciparum
P. vivax
P. knowlesi
T. gondii
BIOCARTA - Regulation of ck1/cdk5 by type 1 glutamate receptors
BIOCARTA - Regulation of eIF2
BIOCARTA - Regulation of eIF4e and p70 S6 Kinase
BIOCARTA - Regulation of hematopoiesis by cytokines
BIOCARTA - Regulation of p27 Phosphorylation during Cell Cycle Progression
BIOCARTA - Regulation of transcriptional activity by PML
BIOCARTA - Repression of Pain Sensation by the Transcriptional Regulator DREAM
BIOCARTA - Reversal of Insulin Resistance by Leptin
BIOCARTA - Rho cell motility signaling pathway
BIOCARTA - Rho-Selective Guanine Exchange Factor AKAP13 Mediates Stress Fiber Formation
BIOCARTA - Role of BRCA1, BRCA2 and ATR in Cancer Susceptibility
BIOCARTA - Role of EGF Receptor Transactivation by GPCRs in Cardiac Hypertrophy
BIOCARTA - Role of Erk5 in Neuronal Survival
BIOCARTA - Role of MEF2D in T-cell Apoptosis
BIOCARTA - Role of Mitochondria in Apoptotic Signaling
BIOCARTA - Role of PI3K subunit p85 in regulation of Actin Organization and Cell Migration
BIOCARTA - Role of PPAR-gamma Coactivators in Obesity and Thermogenesis
BIOCARTA - Role of Parkin in the Ubiquitin-Proteasomal Pathway
BIOCARTA - Role of Tob in T-cell activation
BIOCARTA - Role of nicotinic acetylcholine receptors in the regulation of apoptosis
BIOCARTA - SARS Coronavirus Protease
BIOCARTA - SODD/TNFR1 Signaling Pathway
BIOCARTA - SREBP control of lipid synthesis
BIOCARTA - Selective expression of chemokine receptors during T-cell polarization
BIOCARTA - Shuttle for transfer of acetyl groups from mitochondria to the cytosol

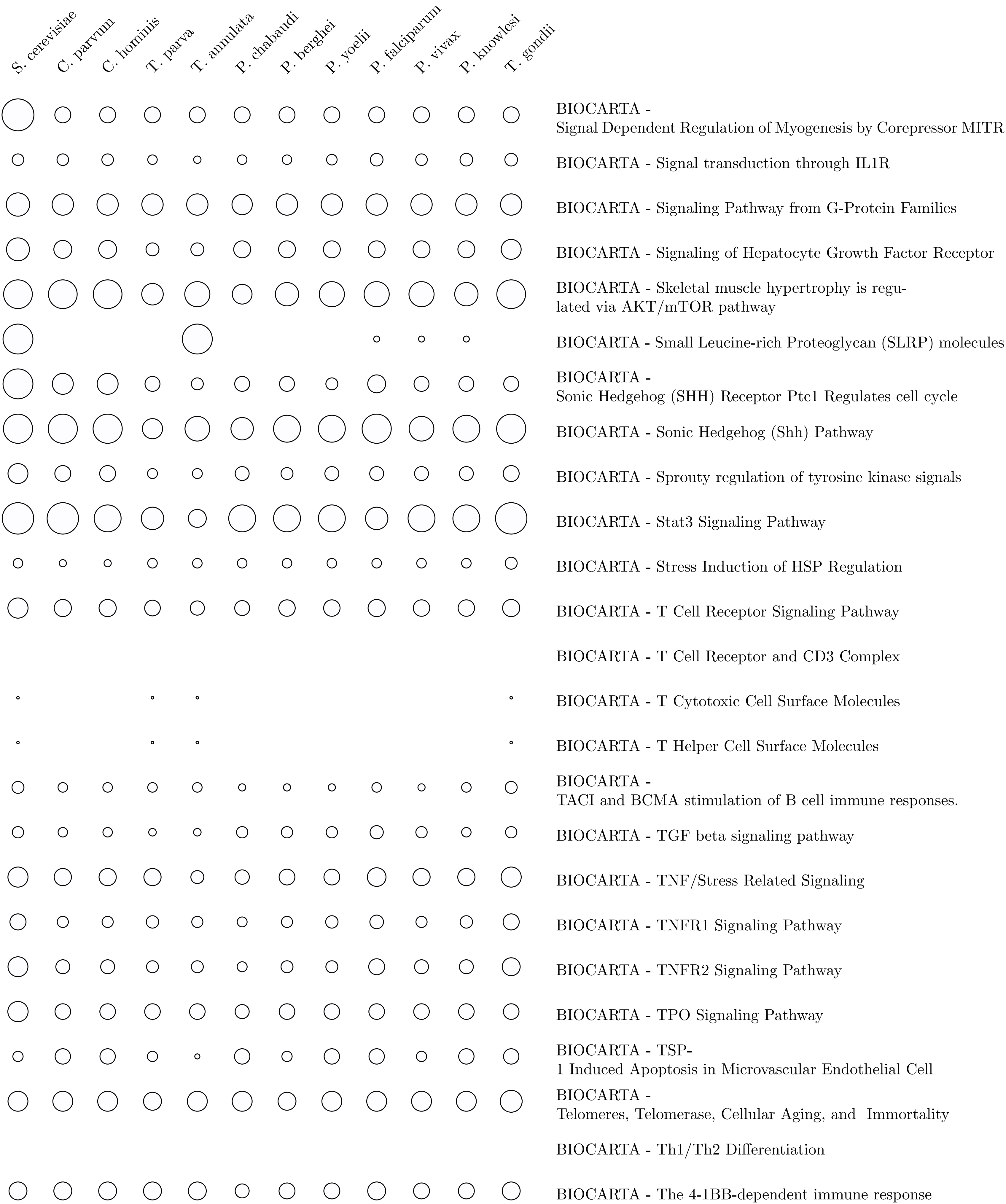
S. cerevisiae
C. parvum
C. hominis
T. parva
T. annulata
P. chabaudi
P. berghei
P. yoelii
P. falciparum
P. vivax
P. knowlesi
T. gondii
BIOCARTA - Signal Dependent Regulation of Myogenesis by Corepressor MITR
BIOCARTA - Signal transduction through IL1R
BIOCARTA - Signaling Pathway from G-Protein Families
BIOCARTA - Signaling of Hepatocyte Growth Factor Receptor
BIOCARTA - Skeletal muscle hypertrophy is regulated via AKT/mTOR pathway
BIOCARTA - Small Leucine-rich Proteoglycan (SLRP) molecules
BIOCARTA - Sonic Hedgehog (SHH) Receptor Ptc1 Regulates cell cycle
BIOCARTA - Sonic Hedgehog (Shh) Pathway
BIOCARTA - Sprouty regulation of tyrosine kinase signals
BIOCARTA - Stat3 Signaling Pathway
BIOCARTA - Stress Induction of HSP Regulation
BIOCARTA - T Cell Receptor Signaling Pathway
BIOCARTA - T Cell Receptor and CD3 Complex
BIOCARTA - T Cytotoxic Cell Surface Molecules
BIOCARTA - T Helper Cell Surface Molecules
BIOCARTA - TACI and BCMA stimulation of B cell immune responses.
BIOCARTA - TGF beta signaling pathway
BIOCARTA - TNF/Stress Related Signaling
BIOCARTA - TNFR1 Signaling Pathway
BIOCARTA - TNFR2 Signaling Pathway
BIOCARTA - TPO Signaling Pathway
BIOCARTA - TSP-1 Induced Apoptosis in Microvascular Endothelial Cell
BIOCARTA - Telomeres, Telomerase, Cellular Aging, and Immortality
BIOCARTA - Th1/Th2 Differentiation
BIOCARTA - The 4-1BB-dependent immune response

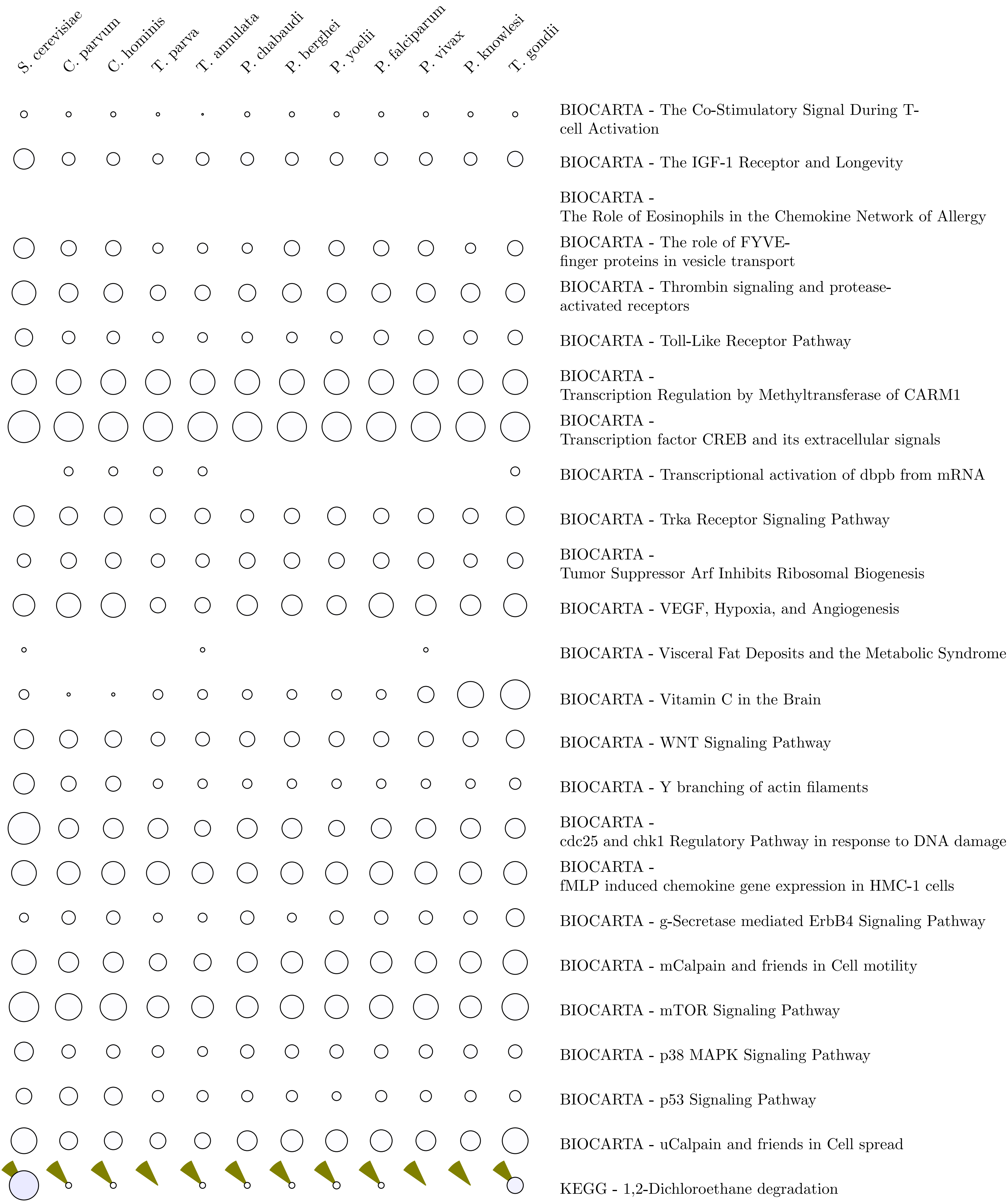
S. cerevisiae
C. parvum
C. hominis
T. parva
T. annulata
P. chabaudi
P. berghei
P. yoelii
P. falciparum
P. vivax
P. knowlesi
T. gondii
BIOCARTA - The Co-Stimulatory Signal During T-cell Activation
BIOCARTA - The IGF-1 Receptor and Longevity
BIOCARTA - The Role of Eosinophils in the Chemokine Network of Allergy
BIOCARTA - The role of FYVE-finger proteins in vesicle transport
BIOCARTA - Thrombin signaling and protease-activated receptors
BIOCARTA - Toll-Like Receptor Pathway
BIOCARTA - Transcription Regulation by Methyltransferase of CARM1
BIOCARTA - Transcription factor CREB and its extracellular signals
BIOCARTA - Transcriptional activation of dbpb from mRNA
BIOCARTA - Trka Receptor Signaling Pathway
BIOCARTA - Tumor Suppressor Arf Inhibits Ribosomal Biogenesis
BIOCARTA - VEGF, Hypoxia, and Angiogenesis
BIOCARTA - Visceral Fat Deposits and the Metabolic Syndrome
BIOCARTA - Vitamin C in the Brain
BIOCARTA - WNT Signaling Pathway
BIOCARTA - Y branching of actin filaments
BIOCARTA - cdc25 and chk1 Regulatory Pathway in response to DNA damage
BIOCARTA - fMLP induced chemokine gene expression in HMC-1 cells
BIOCARTA - g-Secretase mediated ErbB4 Signaling Pathway
BIOCARTA - mCalpain and friends in Cell motility
BIOCARTA - mTOR Signaling Pathway
BIOCARTA - p38 MAPK Signaling Pathway
BIOCARTA - p53 Signaling Pathway
BIOCARTA - uCalpain and friends in Cell spread
KEGG - 1,2-Dichloroethane degradation

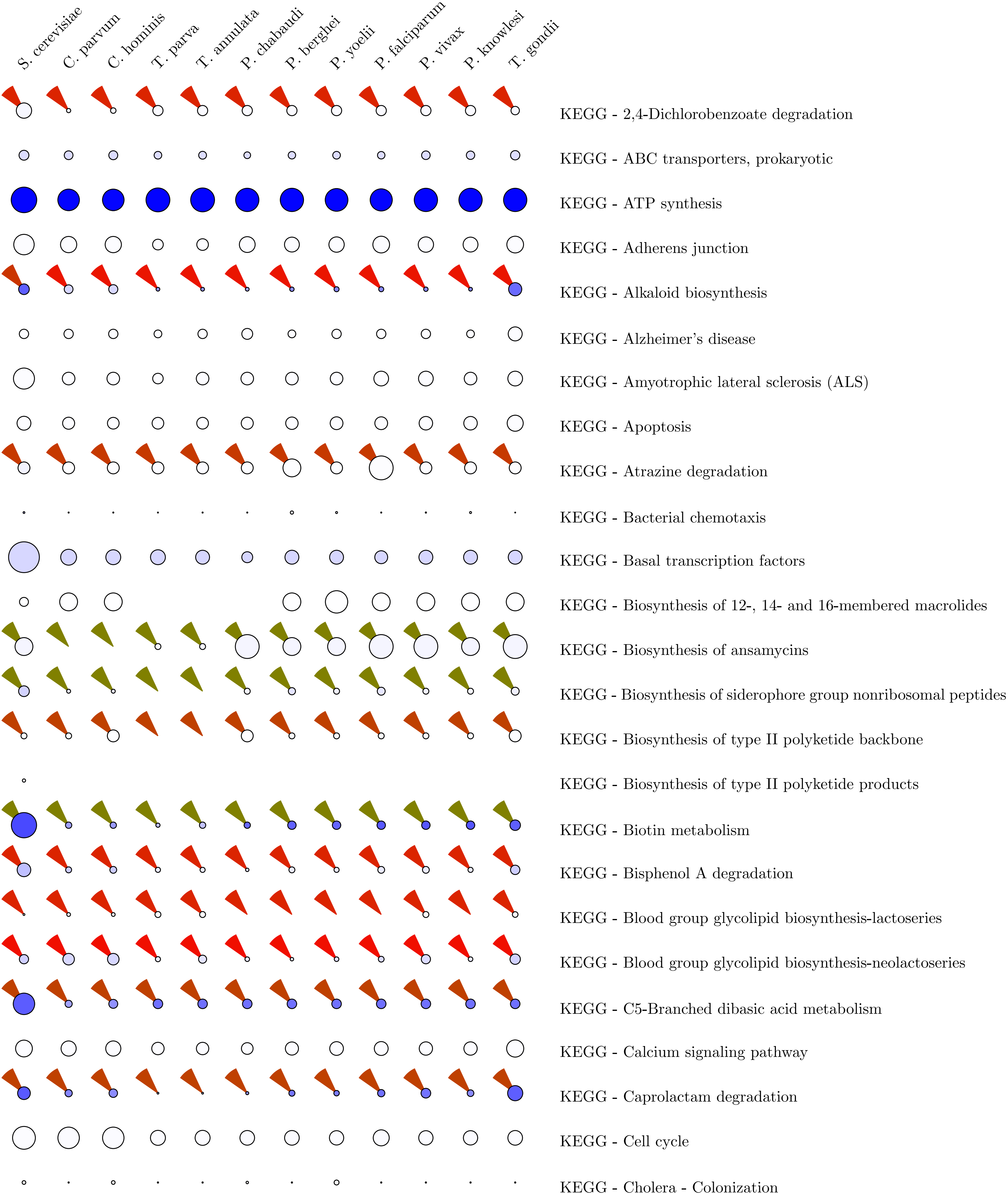
S. cerevisiae
C. parvum
C. hominis
T. parva
T. annulata
P. chabaudi
P. berghei
P. yoelii
P. falciparum
P. vivax
P. knowlesi
T. gondii
KEGG - 2,4-Dichlorobenzoate degradation
KEGG - ABC transporters, prokaryotic
KEGG - ATP synthesis
KEGG - Adherens junction
KEGG - Alkaloid biosynthesis
KEGG - Alzheimer's disease
KEGG - Amyotrophic lateral sclerosis (ALS)
KEGG - Apoptosis
KEGG - Atrazine degradation
KEGG - Bacterial chemotaxis
KEGG - Basal transcription factors
KEGG - Biosynthesis of 12-, 14- and 16-membered macrolides
KEGG - Biosynthesis of ansamycins
KEGG - Biosynthesis of siderophore group nonribosomal peptides
KEGG - Biosynthesis of type II polyketide backbone
KEGG - Biosynthesis of type II polyketide products
KEGG - Biotin metabolism
KEGG - Bisphenol A degradation
KEGG - Blood group glycolipid biosynthesis-lactoseries
KEGG - Blood group glycolipid biosynthesis-neolactoseries
KEGG - C5-Branched dibasic acid metabolism
KEGG - Calcium signaling pathway
KEGG - Caprolactam degradation
KEGG - Cell cycle
KEGG - Cholera - Colonization

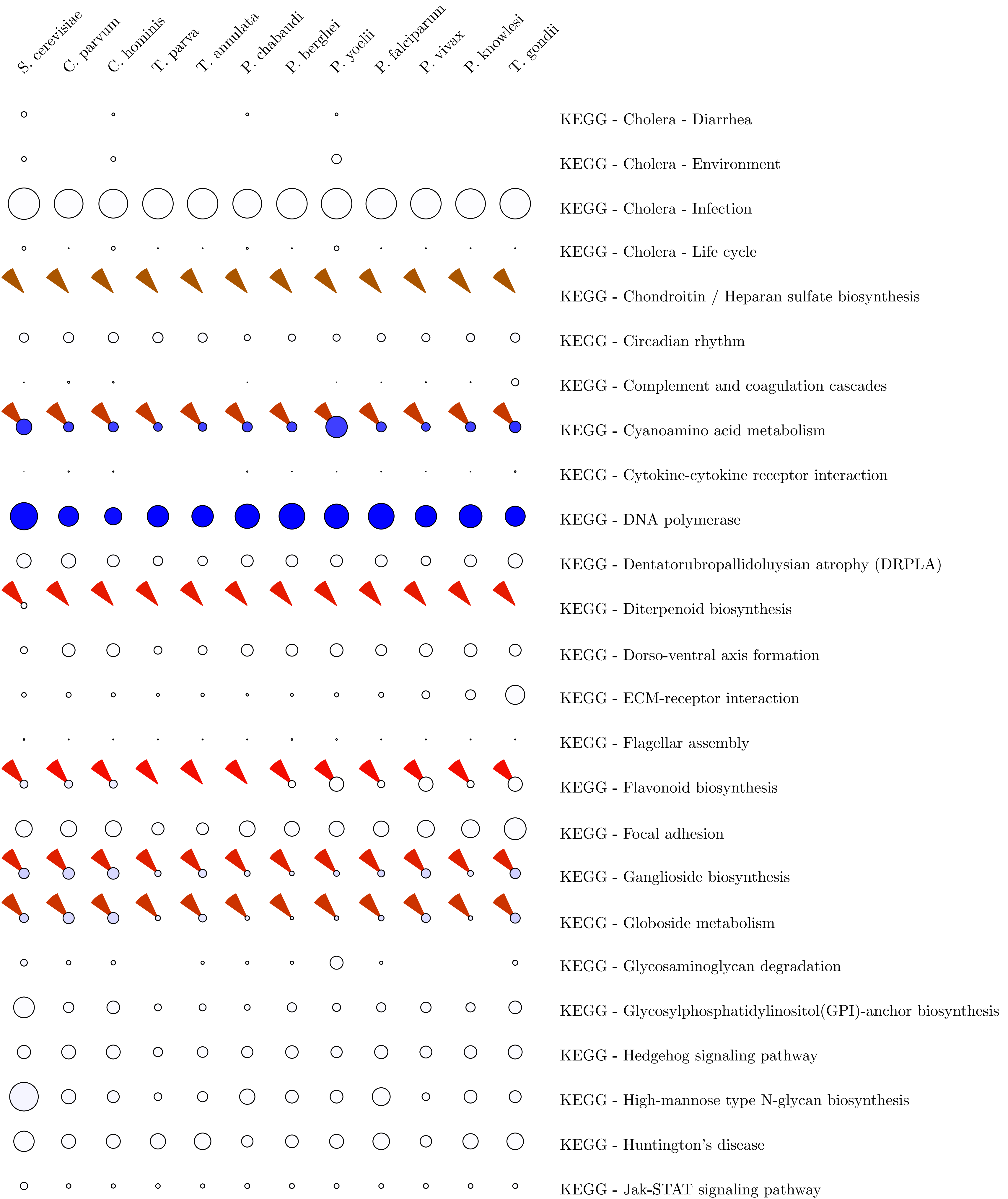

S. cerevisiae
C. parvum
C. hominis
T. parva
T. annulata
P. chabaudi
P. berghei
P. yoelii
P. falciparum
P. vivax
P. knowlesi
T. gondii
KEGG - Cholera - Diarrhea
KEGG - Cholera - Environment
KEGG - Cholera - Infection
KEGG - Cholera - Life cycle
KEGG - Chondroitin / Heparan sulfate biosynthesis
KEGG - Circadian rhythm
KEGG - Complement and coagulation cascades
KEGG - Cyanoamino acid metabolism
KEGG - Cytokine-cytokine receptor interaction
KEGG - DNA polymerase
KEGG - Dentatorubropallidoluysian atrophy (DRPLA)
KEGG - Diterpenoid biosynthesis
KEGG - Dorso-ventral axis formation
KEGG - ECM-receptor interaction
KEGG - Flagellar assembly
KEGG - Flavonoid biosynthesis
KEGG - Focal adhesion
KEGG - Ganglioside biosynthesis
KEGG - Globoside metabolism
KEGG - Glycosaminoglycan degradation
KEGG - Glycosylphosphatidylinositol(GPI)-anchor biosynthesis
KEGG - Hedgehog signaling pathway
KEGG - High-mannose type N-glycan biosynthesis
KEGG - Huntington's disease
KEGG - Jak-STAT signaling pathway

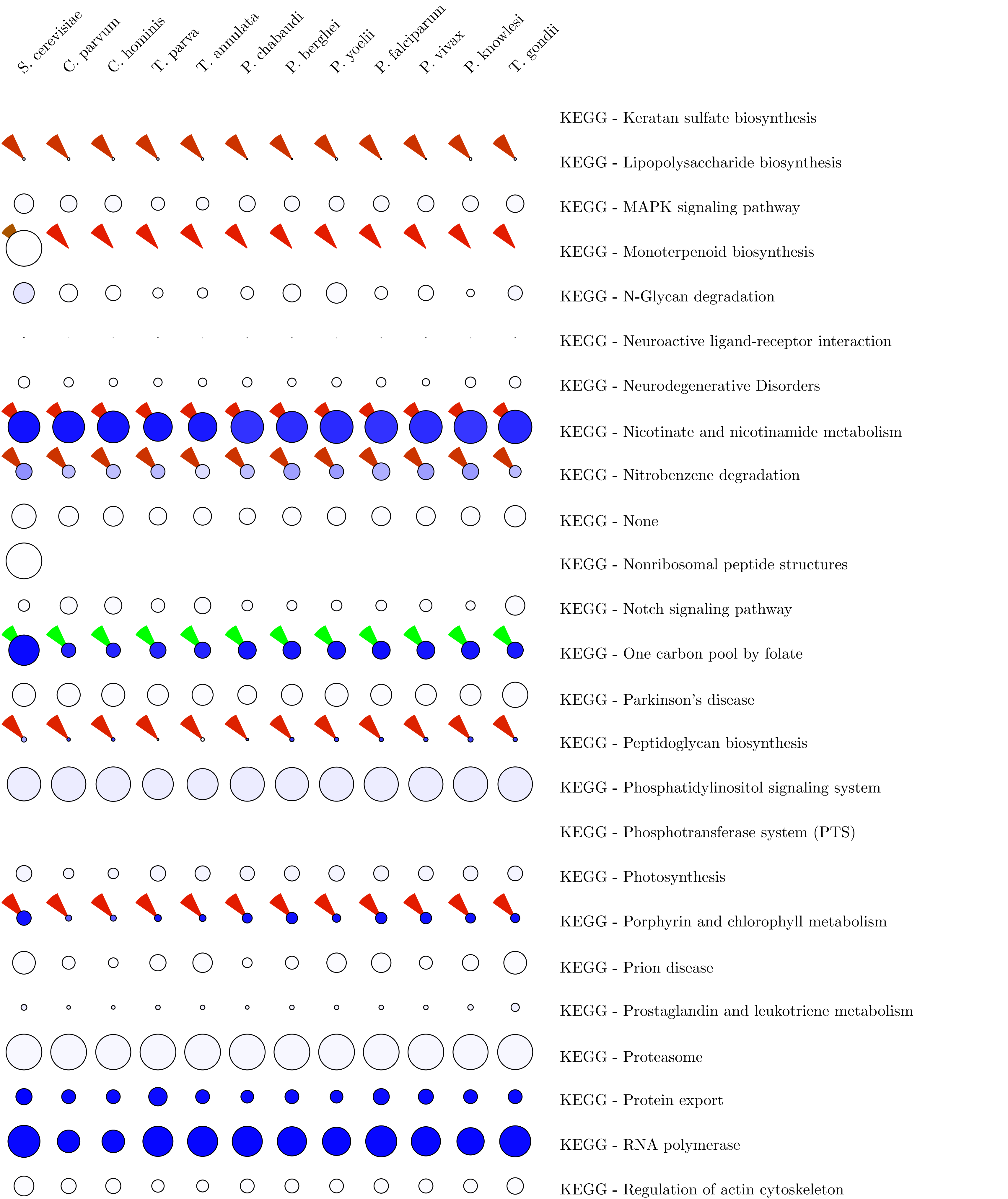
S. cerevisiae
C. parvum
C. hominis
T. parva
T. annulata
P. chabaudi
P. berghei
P. yoelii
P. falciparum
P. vivax
P. knowlesi
T. gondii
KEGG - Keratan sulfate biosynthesis
KEGG - Lipopolysaccharide biosynthesis
KEGG - MAPK signaling pathway
KEGG - Monoterpenoid biosynthesis
KEGG - N-Glycan degradation
KEGG - Neuroactive ligand-receptor interaction
KEGG - Neurodegenerative Disorders
KEGG - Nicotinate and nicotinamide metabolism
KEGG - Nitrobenzene degradation
KEGG - None
KEGG - Nonribosomal peptide structures
KEGG - Notch signaling pathway
KEGG - One carbon pool by folate
KEGG - Parkinson's disease
KEGG - Peptidoglycan biosynthesis
KEGG - Phosphatidylinositol signaling system
KEGG - Phosphotransferase system (PTS)
KEGG - Photosynthesis
KEGG - Porphyrin and chlorophyll metabolism
KEGG - Prion disease
KEGG - Prostaglandin and leukotriene metabolism
KEGG - Proteasome
KEGG - Protein export
KEGG - RNA polymerase
KEGG - Regulation of actin cytoskeleton

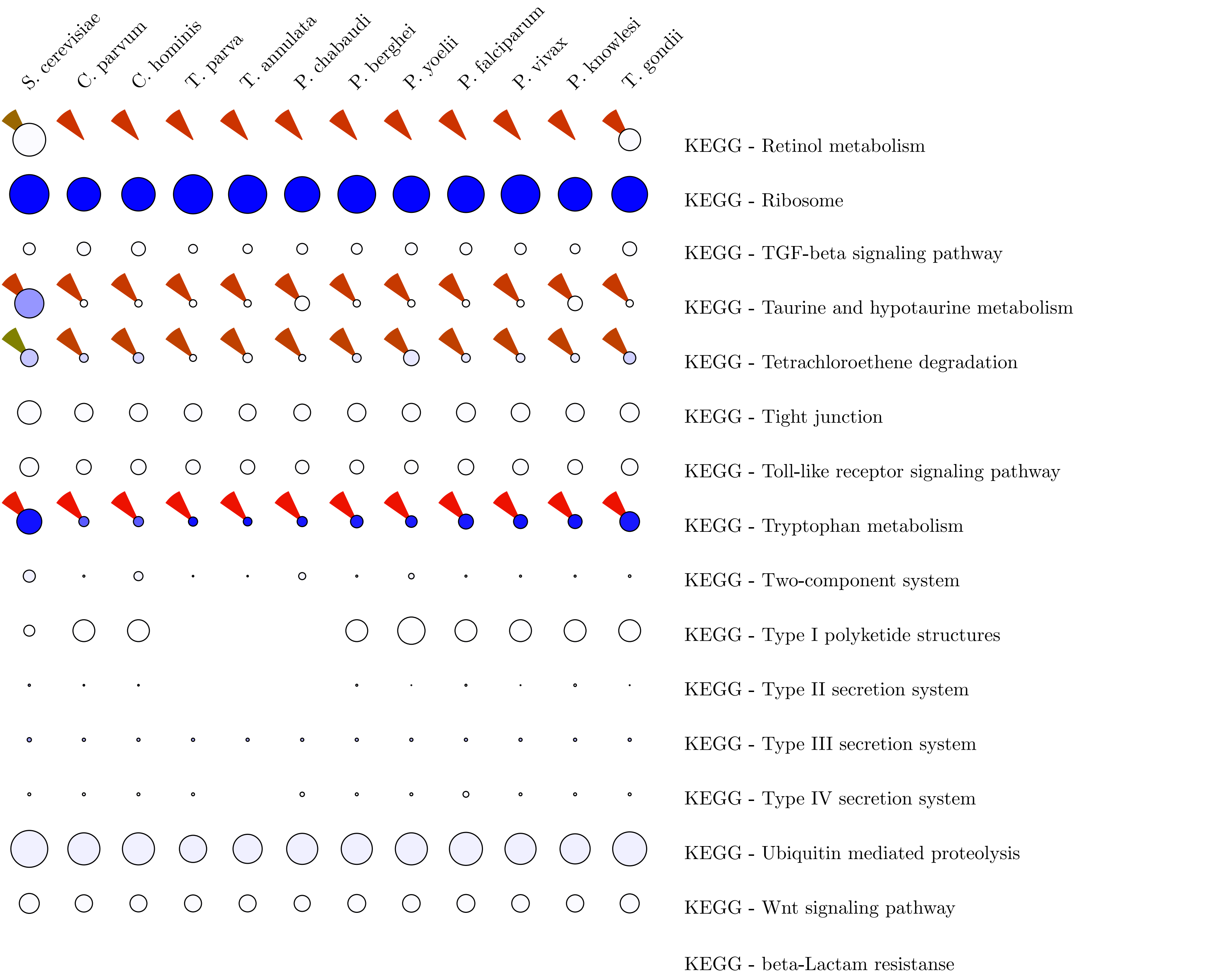
S. cerevisiae
C. parvum
C. hominis
T. parva
T. annulata
P. chabaudi
P. berghei
P. yoelii
P. falciparum
P. vivax
P. knowlesi
T. gondii
KEGG - Retinol metabolism
KEGG - Ribosome
KEGG - TGF-beta signaling pathway
KEGG - Taurine and hypotaurine metabolism
KEGG - Tetrachloroethene degradation
KEGG - Tight junction
KEGG - Toll-like receptor signaling pathway
KEGG - Tryptophan metabolism
KEGG - Two-component system
KEGG - Type I polyketide structures
KEGG - Type II secretion system
KEGG - Type III secretion system
KEGG - Type IV secretion system
KEGG - Ubiquitin mediated proteolysis
KEGG - Wnt signaling pathway
KEGG - beta-Lactam resistanse
