# Supplementary figures and images for: Comparative Genomics of Cryptosporidium
Source: Int J Genomics. 2013 May 2;2013:832756. doi: 10.1155/2013/832756 (PMC3659464; doi:10.1155/2013/832756)

# Genome compactness

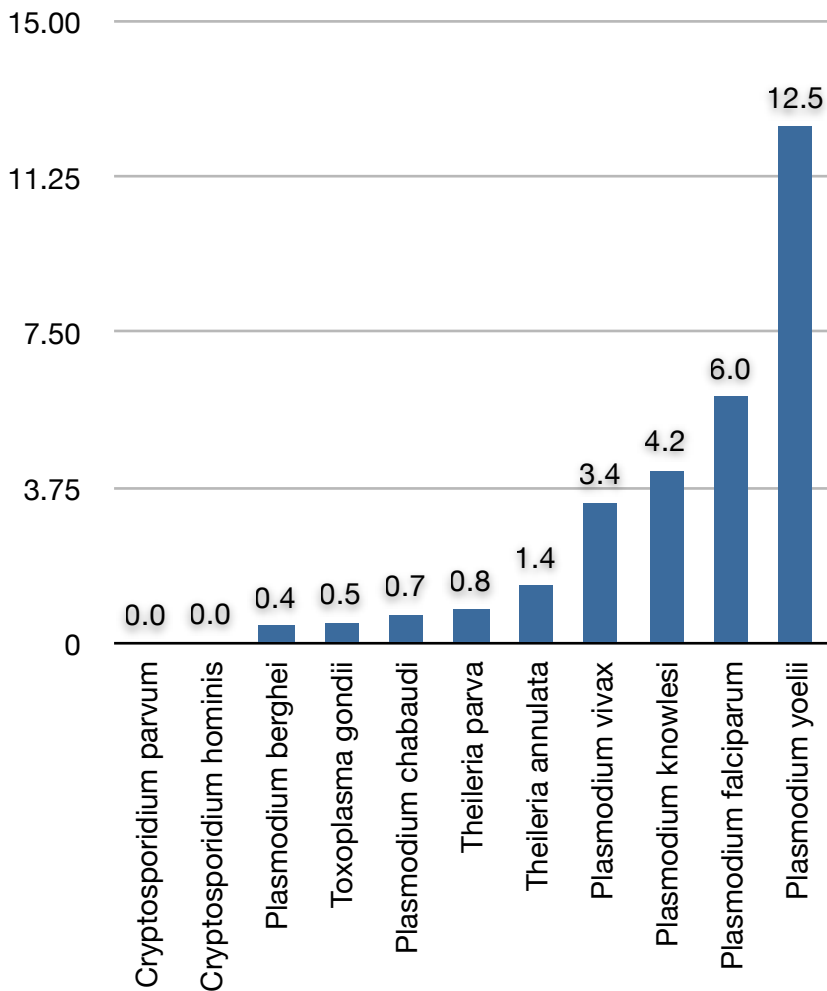

Supplement: Supplementary file 1 — Supplementary Figure 1: Synteny of Plasmodia and Cryptosporidia genus Position of the orthologs identified in the genomes of C. parvum, P. falciparum, P. knowlesi and P. vivax. For each pair of species, a green cross denotes an ortholog present in the same strand in both genomes, while a red cross denotes an inversion. Supplementary Figure 2: Genome Compactness Comparison of the genome compactness of well-studied apicomplexans, as the average number of putative paralogs per genes. Supplementary Figure 3: Pathway Scores See Figure 1. Supplementary Table 1: Chromosomes Composition Results of the statistical evaluation performed using the Fisher's exact test of the enrichment of the Plasmodia chromosomes in orthologs identified in C. parvum. Values of enrichment are given as the log2 of the ratio between the fraction of orthologs in a given Plasmodium chromosome coming from a same chromosome of C. parvum, and the fraction of orthologs in the whole Plasmodium genome coming from this C. parvum chromosome. A value of 0 (ratio of 1) means that the Plasmodium chromosome contains proportionally as many orthologs from the C. parvum chromosome considered as in the whole genome. The values obtained show that the Plasmodium chromosomes significantly retain the composition of the C. parvum chromosomes. P-values below 0.05 were discarded. Supplementary Table 2: Pathway Scores Completeness, connectedness and support scores of the inferred metabolic pathways of C. hominis and C. parvum, along with nine other apicomplexans and an external reference, S. cerevisiae (see text). Supplementary Table 3: Orthologs Sequence Comparison Results of the pairs of putative orthologs sequences comparisons. Identity: percentage of identity between the two protein sequences. dN/dS: non-synonymous on synonymous substitution rate ratio, or None if undefined (when dS equals to zero). Ti/Tv: transition on transversion ratio. Supplementary Table 4: Protein Localizations Predicted protein localization, bas [file 832756.f1.zip › supplementary_figure_2-compactness.pdf]
